# Application of convolutional neural networks towards nuclei segmentation in localization-based super-resolution fluorescence microscopy images

**DOI:** 10.1186/s12859-021-04245-x

**Published:** 2021-06-15

**Authors:** Christopher A. Mela, Yang Liu

**Affiliations:** 1 Department of Biomedical Informatics, University of Pittsburgh, Pittsburgh, PA 15213 USA; 2 Biomedical Optical Imaging Laboratory, Departments of Medicine and Bioengineering, University of Pittsburgh, Pittsburgh, PA 15213 USA

**Keywords:** Super-resolution, STORM, Convolutional neural networks, Mask R-CNN, UNet, StarDist, ANCIS, Nuclei segmentation

## Abstract

**Background:**

Automated segmentation of nuclei in microscopic images has been conducted to enhance throughput in pathological diagnostics and biological research. Segmentation accuracy and speed has been significantly enhanced with the advent of convolutional neural networks. A barrier in the broad application of neural networks to nuclei segmentation is the necessity to train the network using a set of application specific images and image labels. Previous works have attempted to create broadly trained networks for universal nuclei segmentation; however, such networks do not work on all imaging modalities, and best results are still commonly found when the network is retrained on user specific data. Stochastic optical reconstruction microscopy (STORM) based super-resolution fluorescence microscopy has opened a new avenue to image nuclear architecture at nanoscale resolutions. Due to the large size and discontinuous features typical of super-resolution images, automatic nuclei segmentation can be difficult. In this study, we apply commonly used networks (Mask R-CNN and UNet architectures) towards the task of segmenting super-resolution images of nuclei. First, we assess whether networks broadly trained on conventional fluorescence microscopy datasets can accurately segment super-resolution images. Then, we compare the resultant segmentations with results obtained using networks trained directly on our super-resolution data. We next attempt to optimize and compare segmentation accuracy using three different neural network architectures.

**Results:**

Results indicate that super-resolution images are not broadly compatible with neural networks trained on conventional bright-field or fluorescence microscopy images. When the networks were trained on super-resolution data, however, we attained nuclei segmentation accuracies (F1-Score) in excess of 0.8, comparable to past results found when conducting nuclei segmentation on conventional fluorescence microscopy images. Overall, we achieved the best results utilizing the Mask R-CNN architecture.

**Conclusions:**

We found that convolutional neural networks are powerful tools capable of accurately and quickly segmenting localization-based super-resolution microscopy images of nuclei. While broadly trained and widely applicable segmentation algorithms are desirable for quick use with minimal input, optimal results are still found when the network is both trained and tested on visually similar images. We provide a set of Colab notebooks to disseminate the software into the broad scientific community (https://github.com/YangLiuLab/Super-Resolution-Nuclei-Segmentation).

**Supplementary Information:**

The online version contains supplementary material available at 10.1186/s12859-021-04245-x.

## Background

### Introduction

Quantitative analysis of nuclei morphology and architecture plays an important role in understanding nuclear function and assisting pathological diagnosis. Segmentation of cell nuclei is generally the essential first step. Manual segmentation can be highly time-consuming and is impractical for large data sets. Additionally, it suffers from individual user selection bias, boundary errors and lack of reproducibility. Convolutional neural networks (CNNs) have been adapted for nuclei segmentation in wide-field fluorescence images and bright-field histology images with great success [1–3].

With recent advances in super-resolution fluorescence microscopy, nuclear architecture can now be visualized at the molecular scale down to the resolution of 20–30 nm [4–6] in its original spatial context of cells and tissue architecture [7]. In particular, stochastic optical reconstruction microscopy [STORM] has been increasingly used in cell biology to visualize chromatin compaction and spatial relationships between DNA and DNA-binding proteins using various cell models and tissue samples at different pathological stages [5, 6, 8, 9], as they provide significant insights in understanding genome organization and regulation of gene expression important in many fundamental biological processes and diseases. More recently, such approach was applied for examining chromatin structure in clinical tissue samples [7], which may potentially improve cancer diagnosis.

**Fig. 1:**
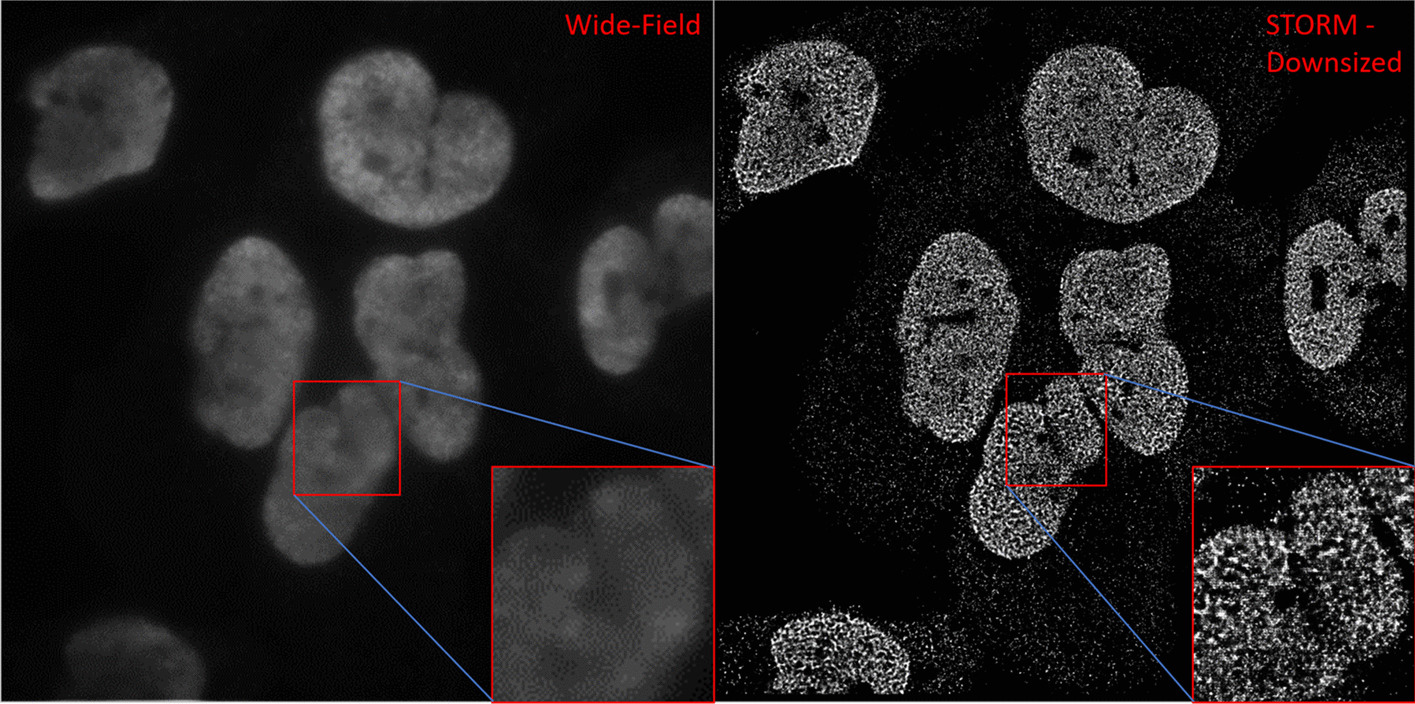
Comparison between conventional wide-field fluorescence microscopy image (left) and STORM super-resolution fluorescence microscopy image (right). Note the differences in nuclei structure between the STORM and wide-field images, with nuclei appearing as collections of discrete packages rather than as contiguous gradients. These textural differences result in incompatibilities when attempting to train a neural network for nuclei segmentation using one data set, then applying the trained network onto the other set

Automated analysis of super-resolution images of cell nuclei has been challenging due to the unique image characteristics distinct from conventional wide-field fluorescence images. Nuclei segmentation on super-resolution images is the essential first step towards quantitative analysis of molecular-scale nuclear architecture and chromatin organization in cells and tissue. The super-resolved nuclear architecture is often discontinuous in nature with frequently disrupted nuclear boundaries (Fig. 1) which may result in partial or over segmentation. Their image size exceeds that of conventional microscopic images by about 100-fold. Further, compared to conventional fluorescence microscopy, localization-based super-resolution microscopy requires ~ 10–20-fold higher illumination laser power (to induce blinking events of the fluorophores for single molecule localization) which can introduce a high background from non-specific binding of the fluorophores and out-of-focus fluorophores. These dense noise regions may be falsely identified as a cell due to the similarity between noise and nuclei regions. In general it has been noted that CNNs tend to produce segmentation results that do not ensure contiguous predictions [10]. Therefore, publicly available CNNs for nuclear segmentation, pre-trained on wide-field fluorescent images, may not be well suited for analyzing super-resolution images.

This study is motivated by the need to identify and segment individual nuclei from localization-based super-resolution images for high-throughput single-cell analysis. Identifying effective neural network architectures for nuclear segmentation and developing optimal image processing algorithms for segmenting localization-based super-resolution images will provide a quick and efficient workflow for quantitative analysis and imaging informatics. In this study we aim to optimize and compare the performance of multiple commonly used network architectures on nuclear segmentation, evaluate the performance of the selected networks when applied to diverse STORM image datasets from cell lines and tissue, and elucidate best practices for nuclear instance segmentation in localization-based super-resolution microscopy images. We will also determine whether networks broadly trained to detect nuclei in a variety of conventional microscopic imagery, such as those used in the 2018 Kaggle Data Science Bowl [1], could be effective in segmenting super-resolution microscopy images, or whether additional training on super-resolution imagery is even necessary.

### Overview of related works

The most cited CNNs for nuclear instance segmentation include UNet [11] and Mask R-CNN [12], which themselves are the basis for many other networks [10, 13–21]. UNet is based on the Fully Convolutional Network (FCN) for semantic segmentation [22, 23], but with the addition of skip connections between the encoder and decoder layers to preserve spatial information and scale [11]. Additionally, UNet does not have any fully connected layers, utilizing a tiling scheme to analyze arbitrarily sized images one tile at a time. Mask R-CNN applies a small CNN for instance segmentation only on the proposed regions generated using Faster R-CNN [12]. Region based approaches limit instance detections to within a bounding box or area of interest, and have become popular techniques for improving segmentation accuracy.

#### Region proposal based segmentation networks

Object detection networks, such as Faster R-CNN [24], Single Shot Detectors (SSD) [25] and You Only Look Once (YOLO) [26], have been implemented in a number of instance segmentation neural networks in addition to Mask R-CNN. Attentive neural cell instance segmentation (ANCIS) applies UNet based segmentation on regions proposed by an SSD [14]. In addition, ANCIS applies two small CNN modules to locate cell features and suppress unwanted noise regions, potentially enhancing accurate delineation of intricate structures and borders. Similarly, the Single Stage Salient-Instance Segmentation network (S4Net) combines a SSD with the segmentation branch of Mask R-CNN [18]. S4Net also utilizes an additional segmentation branch which helps the network to distinguish instances using the contextual information both inside and outside of each proposed region. Another CNN method utilizes Fast YOLO [27] for instance detection and UNet for segmentation [19]. Instance Relation Network (IRNet) builds on Mask R-CNN by adding a small CNN module to each of the region proposal network and segmentation heads [16]. This region proposal module helps to remove duplicate proposals while the segmentation module learns contextual information from the predictions to improve instance differentiation.

#### Non-region proposal based methods in instance segmentation

Alternate to bounding box region proposals, some networks utilize semantic or panoptic segmentation to improve instance segmentation accuracy. Semantic segmentation can be used to identify regions of interest for later instance segmentation [28]. Panoptic segmentation looks to improve instance segmentation by not only identifying target objects of interest, but also by identifying and segmenting out the background and other non-target objects in the image [29].

Various methods are implemented to improve the performance of CNN nuclei segmentation. Among these techniques are edge determination modalities used to better delineate closely grouped or touching nuclei. The original UNet study accomplished this using a loss function that weights edge loss more heavily than interior, forcing the network to better learn instance boundaries [11]. Other techniques include teaching a secondary CNN to learn the borders between clustered cells or the cell edges [21, 30, 31], thresholding, morphological processing, active contours [3] and watershed [32, 33]. These methods are not always trained as part of the network, and are commonly applied during post-processing on the instance predictions made following network training.

Several methods implement a distance transform to aid clustered cell segmentation. One recent technique uses either a UNet or an FCN to predict nuclei locations and estimate boundaries by implementing a regression based learning of the distance maps [34]. Another method uses a trained neural network to predict the Euclidean distance map from the test image, and then applies a Faster R-CNN on the map to localize the individual nuclei [35]. The Hover-Net method learns each prediction’s center of mass, and then applies a sobel operator to determine the horizontal and vertical gradients within each prediction, the results of which were used as inputs into a marker assisted watershed [36]. Lastly, the StarDist method trained an added CNN module to fit star-convex polygons to UNet instance predictions, and then predicted the polygon borders [20]. The accuracy of the border predictions was highly dependent on the image scale.

### Proposed methods

In this study, we aimed to develop an automated and accurate nuclei segmentation tool for localization-based super-resolution images based on convolutional neural networks. Our work specifically addressed nuclei segmentation on the unique characteristic image features of super-resolution images containing heterogeneous discrete features (as opposed to the smoother and more continuous features found in conventional microscopy images), noisy background and large image size.

We performed extensive evaluation of the performance of CNN architectures towards instance segmentation of nuclei from STORM-based super-resolution images. First, we evaluated whether networks broadly trained on publicly available nuclear images adopted from the 2018 Kaggle Data Bowl competition [1] could be directly applied towards the nuclear segmentation of our super-resolution image dataset. We then trained, optimized and tested each of three selected CNNs exclusively on our super-resolution image datasets. Our STORM images of cell nuclei include a diverse spatial “texture”, labeled with fluorophores attached to various molecular targets, from different cell and tissue types, biological states and pathological states. For each network, we evaluated the effect of various pre- and post-processing methods (such as downsizing, blurring, contrast enhancement) of STORM images on test accuracy. Lastly, we developed a noise removal approach to improve instance segmentation on super-resolution images and the effect of overlapping regions on test accuracy.

## Results

Results indicate that super-resolution images are not broadly compatible with neural networks trained on conventional bright-field or fluorescence microscopy images. When the networks were trained on super-resolution data, however, we attained nuclei segmentation accuracies (F1-Score) in excess of 0.8, comparable to past results found when conducting nuclei segmentation on conventional fluorescence microscopy images. Overall, we achieved the best results utilizing the Mask R-CNN architecture.

Three convolutional neural networks were selected for training and testing. We focused initially on broadly used networks for image segmentation, particularly UNet and Mask R-CNN [11, 12]. UNet, however, inherently performs semantic segmentation. Therefore, we instead selected a UNet-based instance segmentation algorithm, StarDist [20]. To evaluate the effect of region proposal networks using the same base segmentation network (UNet), we selected ANCIS [14]. In addition, this selection allowed us to compare two region-based localization methods with different segmentation algorithms in ANCIS and Mask R-CNN. The segmentation results from each technique were compared. Additionally, we tested various image pre and post processing techniques, and train a UNet for noise detection and removal from our test images.

### Initial testing

**Fig. 2:**
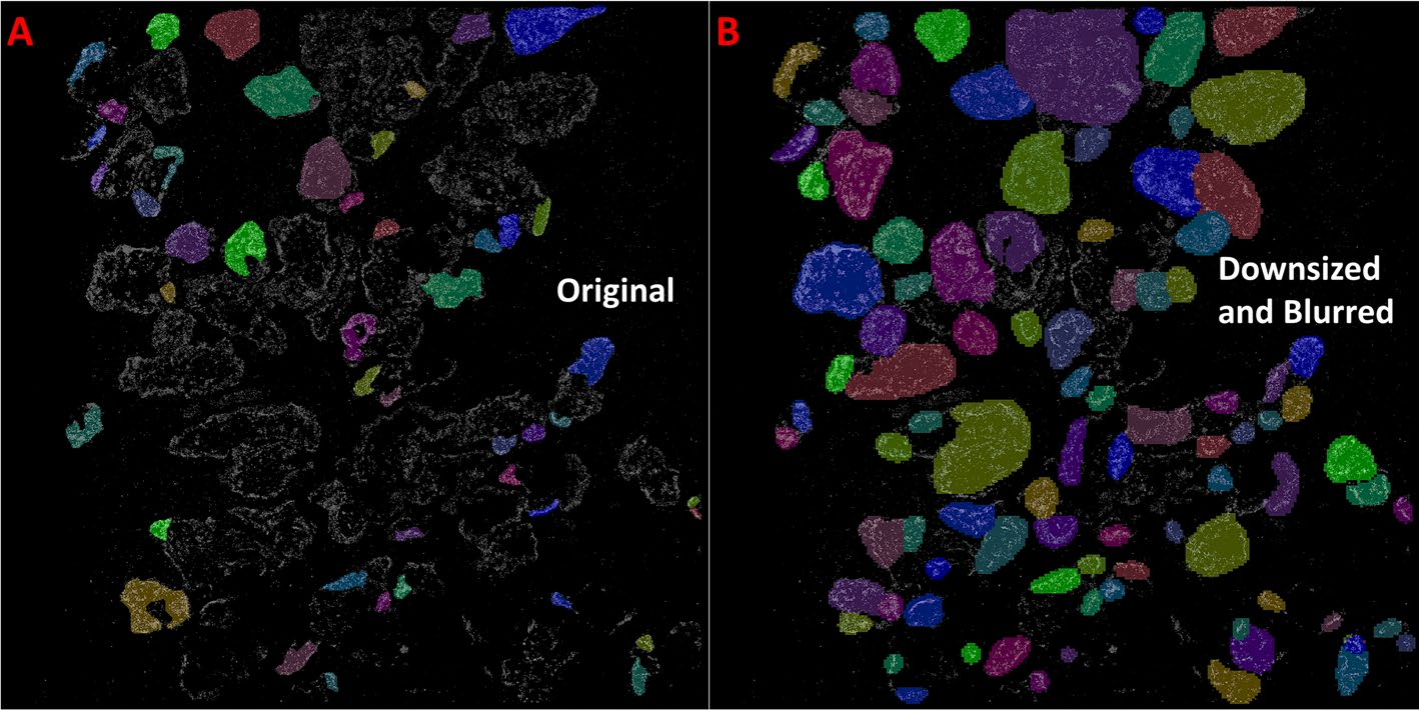
Segmentation results using a Kaggle dataset trained Mask R-CNN applied to our STORM images. Segmented super-resolution images from the colon tissue dataset were originally downsized to 512 × 512 before testing (**a**), and then further downsized to 256 × 256 and blurred before segmenting again (**b**). The Kaggle trained network attained superior results on the blurred 256 × 256 sized image, however still demonstrated significant over segmentation. This result demonstrates the need to train these CNN segmentation methods on the super-resolution data directly

We first trained our networks using the broad, publicly available Kaggle dataset [1]. The Kaggle dataset includes a large set of 2D microscopic images of cell nuclei from cell culture and tissue across various imaging modalities including conventional fluorescence, histology and bright-field microscopy. Although super-resolution fluorescence microscopy is a type of fluorescence microscopy, super-resolved images may or may not prove compatible with CNNs trained using traditional fluorescence microscopy images. Our goal here is to determine whether training directly on a super-resolution STORM image dataset is necessary. Each network was trained using the optimal number of epochs, steps and other factors determined by the authors of each method. Trained networks were then applied to our resized STORM images from 512 × 512 tissue and cell line test sets. As shown in Fig. 2A and Table 1, the optimal results were achieved using Mask R-CNN, but the overall performance is poor. The F1 score on the tissue and cell line dataset is only 0.181 and 0.073, respectively.

**Table 1:** Average test accuracy scores for Mask-RCNN trained on Kaggle dataset and tested on super-resolution imagery

Test set	Pre-processing	F1-Score	FN	Hausdorff
Colon tissue	512 × 512	0.181	0.819	14.07
	256 × 256	0.268	0.619	8.68
	256 × 256 Blur	0.262	0.635	8.03
	256 × 256 HEq	0.352	0.501	8.22
Cell line	512 × 512	0.073	0.924	12.83
	256 × 256	0.475	0.473	6.9
	256 × 256 Blur	0.555	0.268	5.92
	256 × 256 HEq	0.628	0.201	6.07

Average F1-Score, false negative percent (FN) and Hausdorff distance for a Mask R-CNN segmentation network model trained on the Kaggle dataset, and applied to both our super-resolution colon tissue and DNA labelled cell line datasets. The network was applied to our Colon Tissue and Cell Line image test sets (512 × 512 resolution), as well as to the downsized versions of each test set (256 × 256 resolution), and to Gaussian blurred (Blur) and histogram equalized (HEq) versions

Next, we applied a set of pre-processing methods to improve the performance. The STORM images have two unique aspects compared to conventional fluorescence images: the inherently discontinuous structural features at the nearly molecular-scale resolution and the nearly zero “intensity” value in most background regions. We deliberately lowered the image resolution by downsizing to 256 × 256 and either blurring or altering the image contrast by histogram equalization (Fig. 2B). These pre-processing methods overall did improve our test accuracy, while decreasing the false negative percentile (Table 1). However, none of the Kaggle trained models succeeded in attaining an average F1-Score exceeding 0.5 on the tissue dataset, and a top mark of 0.628 was achieved on the cell line dataset. Notable exceptions to these results can be found when analyzing individual images. Kaggle trained Mask R-CNN performed markedly better on STORM images containing dense or uniform nuclear texture with clear borders in both cell line and tissue images (Additional file 1: Figure S1). The F1-scores of these segmentations were found to be less than those found using the STORM-trained Mask R-CNN network, but they demonstrate the potential of CNNs towards the segmentation of super-resolution images.

### Optimization

Due to the generally poor results when applying the Kaggle trained networks, we next trained each network directly on our STORM image datasets. We determined parameters for instance segmentation via a process of training and testing, and utilized test accuracy as the determinant factor for best performance. Test accuracy, for optimization purposes, was assessed using the F1-Score calculation at an IoU threshold of 0.7. Parameters varied included number of epochs and number of training images for all networks, as well as number of steps for Mask R-CNN and StarDist. Additional parameters, included learning rate and other network specific variables, were optimized as well. A summary of optimal parameters determined herein can be found in Table 2.

**Table 2:** Summary of optimal training parameters determined using each network for each training set

DataSet	Optimal parameters	Network		
		Mask R-CNN	Stardist	ANCIS
Tissue	Epochs	400	400	200 (500 RPN)
	Steps	500	300	NA
	Learning Rate	1E−4	1E−4	1E−4
	Training Set	77*	77*	77*
Cell	Epochs	600	400	400 (500 RPN)
	Steps	500	300	NA
	Learning Rate	1E−4	1E−4	1E−4
	Training Set	60	65*	65*

^*^Indicates the number is the maximum number of images available in the dataset

#### Tissue dataset

**Fig. 3:**
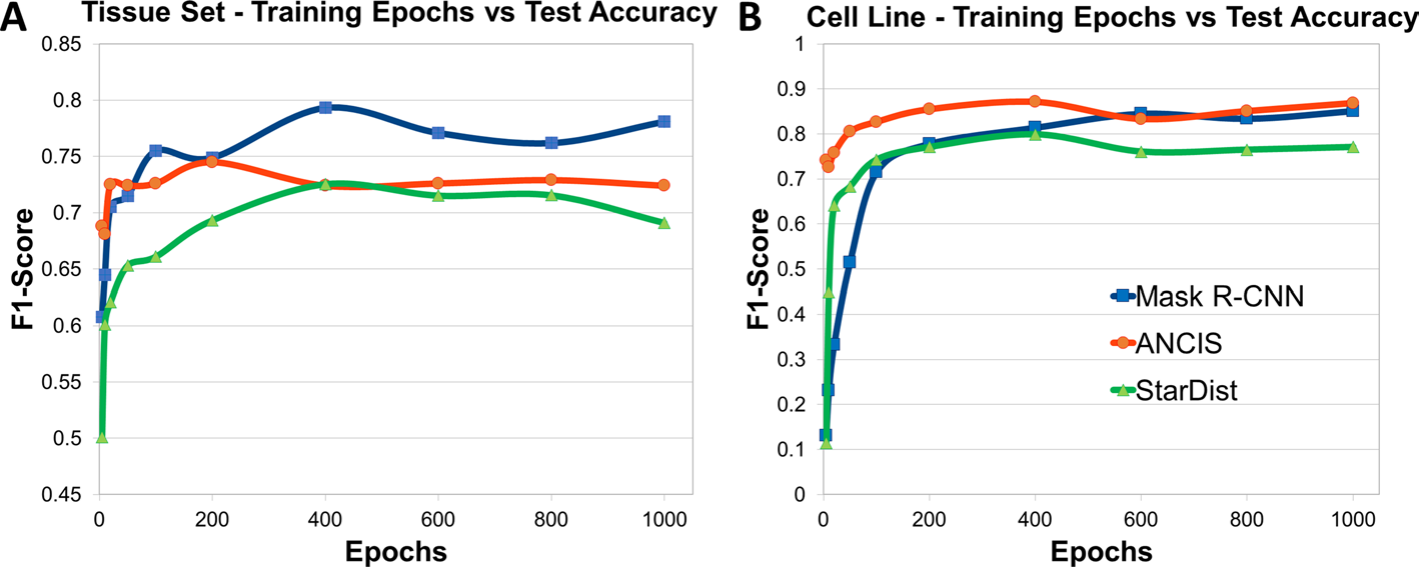
Test accuracy as a function of F1-Score versus number of training epochs. Optimization plots depicting F1-Score versus number of training epochs for each network model trained on the colon tissue dataset (**a**), or on the cell line A dataset (**b**)

A typical trend observed was a quick rise in accuracy with increasing number of epochs, followed by a fluctuation and then a settling. After a larger number of epochs, network accuracy was observed to level or drop, likely due to overfitting (Fig. 3A). Plotting the test accuracy against number of steps provided a similar trend, with networks reaching an accuracy saturation point when using more than a few hundred steps. Note that ANCIS did not provide the ability to vary steps, but rather utilized a two-part training conducted first on the region-based localization network, then on the instance segmentation network.

It was observed that for Mask R-CNN and StarDist, the optimal number of epochs occurred at about 400, whereas 200 epochs produced the peak F1-Score for ANCIS. Both UNet methods, ANCIS and StarDist, showed some decrease in test accuracy with increasing epochs, beyond the optimal range. Mask R-CNN, on the other hand, appeared to fluctuate. Increasing steps improved the accuracy of Mask R-CNN more linearly, beyond 100 steps, without significant fluctuation until beyond 500 steps, suggesting nonlinear effects when using too many or too few epochs (Additional file 2: Figure S2). StarDist reached a peak accuracy at 300 steps, then dropped off, as with increasing epochs, perhaps due to overfitting.

Increasing the size of the training set was expected to improve test accuracy, and this was generally found to be the case (Additional file 3: Figure S3). All networks improved significantly when increasing from 10 to 20 training images, with each image containing an average of 22 instances. ANCIS and StarDist continued to improve at a nearly linear trend beyond 20 images, however Mask R-CNN once again demonstrated a fluctuating trend. All networks performed best when using the entire available training set of 77 images, however satisfactory results could be obtained using less. False negative or false positive counts tended to be higher when using a smaller dataset, and overlapping detections occurred with greater frequency.

Varying the learning rate within a limited, though often used, range of 1e−3 to 1e−5 did not produce a great deviation in test accuracy, however a lower learning rate tended to require more epochs to achieve the same accuracy. Since all learning rates within this range provided similar results, we selected a rate in the middle of the range, resulting in a common rate of 1e−4 for all networks.

#### Cell line dataset

The trend for test accuracy versus number of epochs for the cell line data proved to be similar to the trend to the results from the tissue dataset, however the F1-Score values were higher overall with less fluctuation (Fig. 3B). More uniform shapes, less noise and greater spacing in-between instances (i.e. less clustering) may help account for the increased accuracy for the nuclei segmentation on the cell line dataset versus the tissue dataset. The smoother accuracy-versus-epoch curves may also be accounted for by the reduced variability between the target instances. The optimal number of epochs, steps and learning rate used were found to also be similar to those from the tissue dataset, but not the same. Optimal number of epochs were determined at 400 for both StarDist and ANCIS, but 600 for Mask R-CNN. Steps versus test accuracy, however, progressed similarly to the results found for the tissue training set.

Effect of training set size was also determined for the cell line dataset consisting of 65 training images. The performances were overall improved for all networks with increasing dataset size (Additional file 3: Figure S3), although Mask R-CNN dropped in accuracy when using the entire dataset, from 0.869 to 0.831. Both ANCIS and StarDist fluctuated between 40 and 60 images, but maintained an overall upward trend in images versus accuracy. ANCIS demonstrated both the highest scores and least variation. Indeed, the F1-Score for ANCIS when trained on only 10 images was nearly 0.9, with each image containing an average of 4.2 nuclei per image. However, the false negative percent of this model was much higher than the model trained on the full dataset, 12.5% for the former and 3% the latter, as was the Hausdorff distance, 8.53 and 6.39 respectively. Mask R-CNN demonstrated a similar trend, scoring fairly high even when only trained on 10 images, though scores were not as high as for the ANCIS model.

### Network testing

**Fig. 4:**
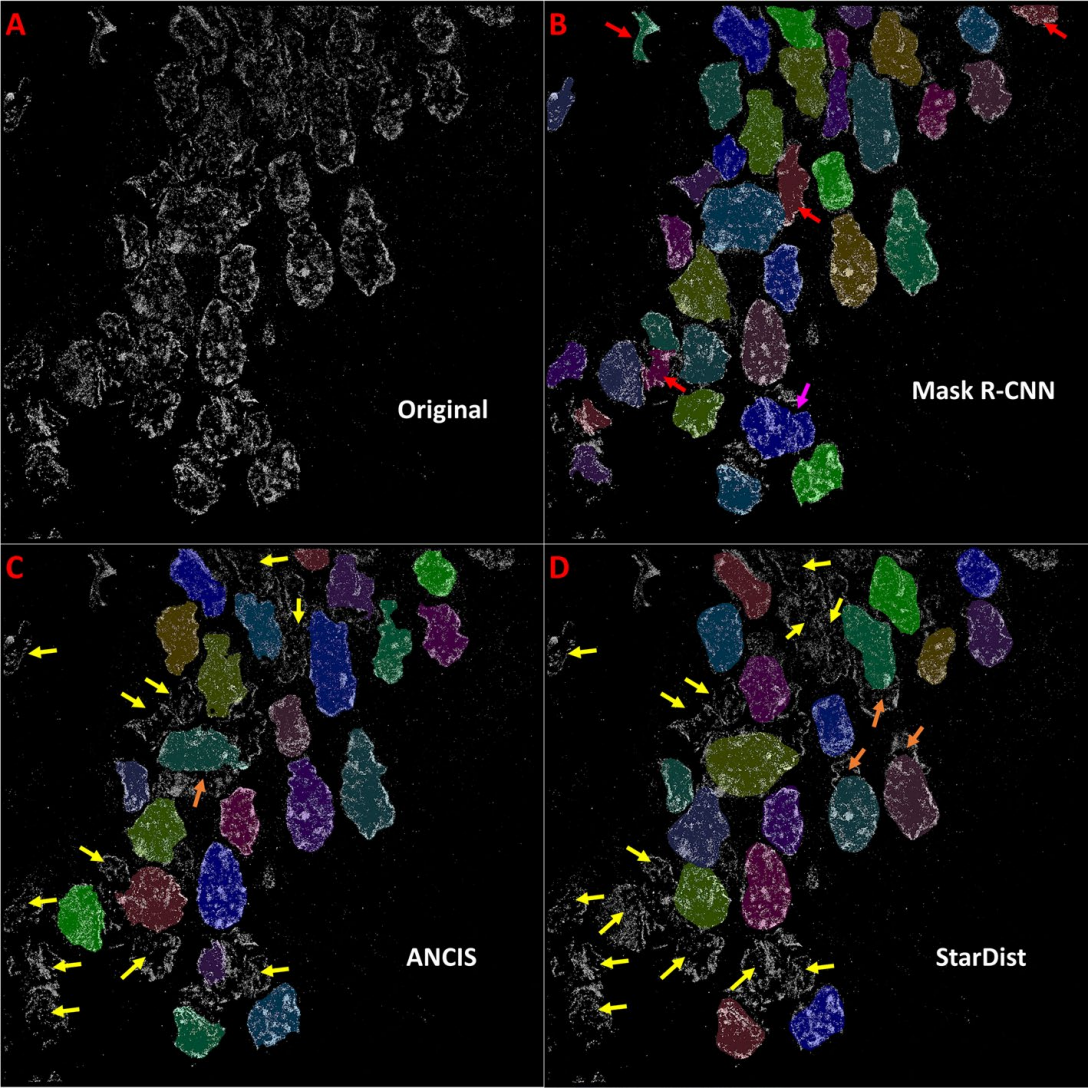
Segmentation examples for a colon tissue image (H3K9me3) depicting a high-grade dysplasia. Original test image (**a**) is segmented using (**b**) Mask R-CNN, (**c**) ANCIS and (**d**) StarDist network models. Training and testing were done on colon tissue data. Red arrows indicate False Positives, yellow arrows point out False Negatives, and pink and orange arrows indicate over- and under-segmentations, respectively

Following network training and optimization, nuclei segmentation was conducted on all test image sets (Fig. 4). The tissue dataset included the STORM images of nuclei labeled with a heterochromatin marker H3K9me3 from both colon and prostate tissue at different pathological states (normal, low-grade and high-grade pre-cancerous lesions and invasive cancer). When evaluating the cell line dataset, the test set included images with various labeled molecular targets (H3K27me3, H3K4me3, DNA, RNA polymerase II) from different cell lines under normal and treated conditions. Test accuracy was assessed again using the F1-Score of instances that achieved an IoU of 0.7. Additionally, we calculated the percentage of False Negatives and the average Hausdorff Distance, to provide an estimate of border and instance positioning accuracy.

#### Tissue dataset

STORM images of colon tissue dataset include various pathological states including normal tissue, precancerous (adenoma, high-grade dysplasia) and invasive cancer. Nuclear texture for different pathological phenotype varies significantly, where the nuclear texture from precancerous (adenoma and high-grade dysplasia) and cancerous tissue exhibit dramatically fragmented chromatin texture with highly disrupted borders. Comparing network performance when trained and tested on the colon tissue dataset, we found that the Mask R-CNN model provided the highest test accuracy (Table 3). All networks demonstrated an optimal F1 score of at least 0.72 on the tissue dataset, but none achieved a score greater than 0.80. Additionally, Mask R-CNN was found to result in the lowest average Hausdorff distance, implying the greatest average instance border accuracy. Mask R-CNN also demonstrated the lowest false negative rate, but also had the highest number of false positives, suggesting some degree of over-segmentation. ANCIS, on the other hand, had the highest false negative rate and lowest number of false positives, suggesting under-segmentation (Fig. 4). StarDist performed similarly to ANCIS, although with slightly lower accuracy, less false negatives and higher Hausdorff distance.

**Table 3:** Average test accuracy scores for CNNs trained and tested on super-resolution imagery

DataSet		Mask R-CNN			ANCIS			StarDist		
Train set	Test set	F1	FN/FP	H	F1	FN/FP	H	F1	FN/FP	H
Colon tissue	Colon	**0.793**	**0.177**/0.225	**9.76**	0.739	0.315/**0.122**	10.68	0.725	0.253/0.126	10.62
	Prostate	0.646	**0.221**/0.121	**8.82**	0.505	0.496/**0.118**	9.64	**0.673**	0.357/0.071	9.17
	Cell downsize	**0.872**	**0.053**/0.17	**5.65**	0.601	0.201/**0.134**	13.21	0.847	0.137/0.105	6.39
Cell Line A (discrete)	Cell line	0.832	0.11/0.3	8.21	**0.902**	**0.101**/**0.078**	**7.05**	0.799	0.107/0.263	8.87
Cell Line B (all textures)	Cell line	0.831	0.125/**0.116**	7.91	**0.952**	**0.03**/0.128	**6.39**	0.859	0.076/0.31	8.11
	Colon upsize	0.423	0.607/0.424	17.43	**0.489**	0.551/**0.076**	**14.51**	0.39	**0.467**/0.645	17.06
Combined (Colon & Cell)	Colon	0.676	**0.169**/0.564	**10.59**	**0.753**	0.305/**0.11**	11.13	0.612	0.396/0.397	11.39
	Cell line	0.885	**0.021**/0.236	5.54	**0.943**	0.041/**0.107**	6.76	0.92	0.037/0.174	**5.45**
Colon Blur	Colon blur	**0.752**	**0.246**/0.25	**9.87**	0.729	0.349/**0.136**	11.06	0.658	0.387/0.141	11.02
Colon HEQ	Colon HEQ	**0.769**	**0.183**/0.287	**10.01**	0.733	0.328/0.127	11.17	0.696	0.348/**0.123**	10.94
Cell A Blur	Cell blur	0.791	0.12/0.333	6.87	**0.858**	**0.112**/**0.118**	**6.83**	0.761	0.153/0.288	9.22
Cell A HEQ	Cell HEQ	0.81	0.105/0.363	6.64	**0.867**	**0.098**/**0.126**	**6.57**	0.785	0.14/0.285	8.99

F1-Score (**F1**), false negative percent and false positive percent (**FN/FP**), and Hausdorff distance (**H**) for Mask R-CNN, ANCIS and StarDist network models trained on the STORM colon tissue dataset, and cell line datasets A & B. An additional combined training dataset included downsized cell line dataset A, colon tissue and Kaggle datasets. Testing was conducted on the 512 × 512 colon and prostate tissue test sets as well as on the 512 × 512 cell line test set, downsized (256 × 256) cell line test set and upsized (1024 × 1024) colon test set. Training and testing were also conducted on histogram equalized (HEQ) and blurred (Blur) versions of the colon tissue and cell line A datasets. Pre-processing was conducted on the 512 × 512 versions of the colon tissue and cell line A datasets for both the training and test images. The results indicate that the original data provided the best test accuracy over the pre-processed images for all cases, suggesting no advantage to be gained by these processes. Top results for each test set are indicated by **bold** numbering

Further, we evaluated whether the networks trained on the dataset from one type of biological sample (e.g., cell line dataset) can be directly used on another type (e.g., tissue dataset) to determine cross compatibility between trained models. We first tested the networks trained using cell line A data on the colon tissue images. Accuracy scores were found to be low, but improved somewhat when the test tissue images were resized to 1024 × 1024 (i.e. upsized to make the tissue nuclei similar in size to those of the cell line). False negatives and Hausdorff distances were also much higher than when segmenting with a tissue-trained model. Generally, the models trained with cell line dataset did not perform well when applied to tissue images. We then tested networks trained using the colon tissue data on the cell line test set. Again, initial results were poor (resulting in extensive over-segmentation), but improved drastically when the test set was downsized to 256 × 256 (in order to make the cell nuclei similar in size to those of the colon tissue images). Downsizing worked better for the Mask R-CNN and Stardist networks than for ANCIS (Table 3).

We also briefly compared results between normal nuclei and those at different pathological states within our colon tissue test set. Segmentation test accuracy was found to be significantly better on the normal nuclear phenotypes (F1 = 0.919) than on the pathological phenotypes (low-grade F1 = 0.825, high-grade F1 = 0.779 and invasive adenocarcinoma F1 = 0.676), when training on the STORM colon tissue dataset using Mask R-CNN (Additional file 4: Figure S4). Scores for ANCIS (normal F1 = 0.871, low-grade F1 = 0.783, high-grade F1 = 0.676 and invasive F1 = 0.653) and StarDist (normal F1 = 0.895, low-grade F1 = 0.702, high-grade F1 = 0.678 and invasive F1 = 0.532) proceeded similarly. Enhanced performance on the normal tissue is likely due to the dense nuclear texture and more well-spaced nuclei observed in those images, compared to the more clustered and irregular nuclei found and disrupted nuclear texture in pathological tissue sample images.

Lastly, we evaluated the cross compatibility between different tissue types. We applied the models trained on the original colon tissue dataset trained networks to the prostate tissue test set, labeled with the same nuclear marker (H3K9me3), expressing multiple pathological phenotypes (normal, low-grade and high-grade prostatic intraepithelial neoplasia and invasive cancer). The network segmentation was found to be acceptable across phenotypes (F1 = 0.646, Mask R-CNN), but the accuracy was significantly lower than for the segmentation on the colon tissue test set (F1 = 0.793) (Additional file 5: Figure S5). Potential causes for the reduced accuracy were the variations in nuclear shape (more circular) and texture (consisting of more discrete fragments) when compared to the colon tissue nuclei. The prostate tissue images also contained a denser noise in between nuclei.

#### Cell line dataset

**Fig. 5:**
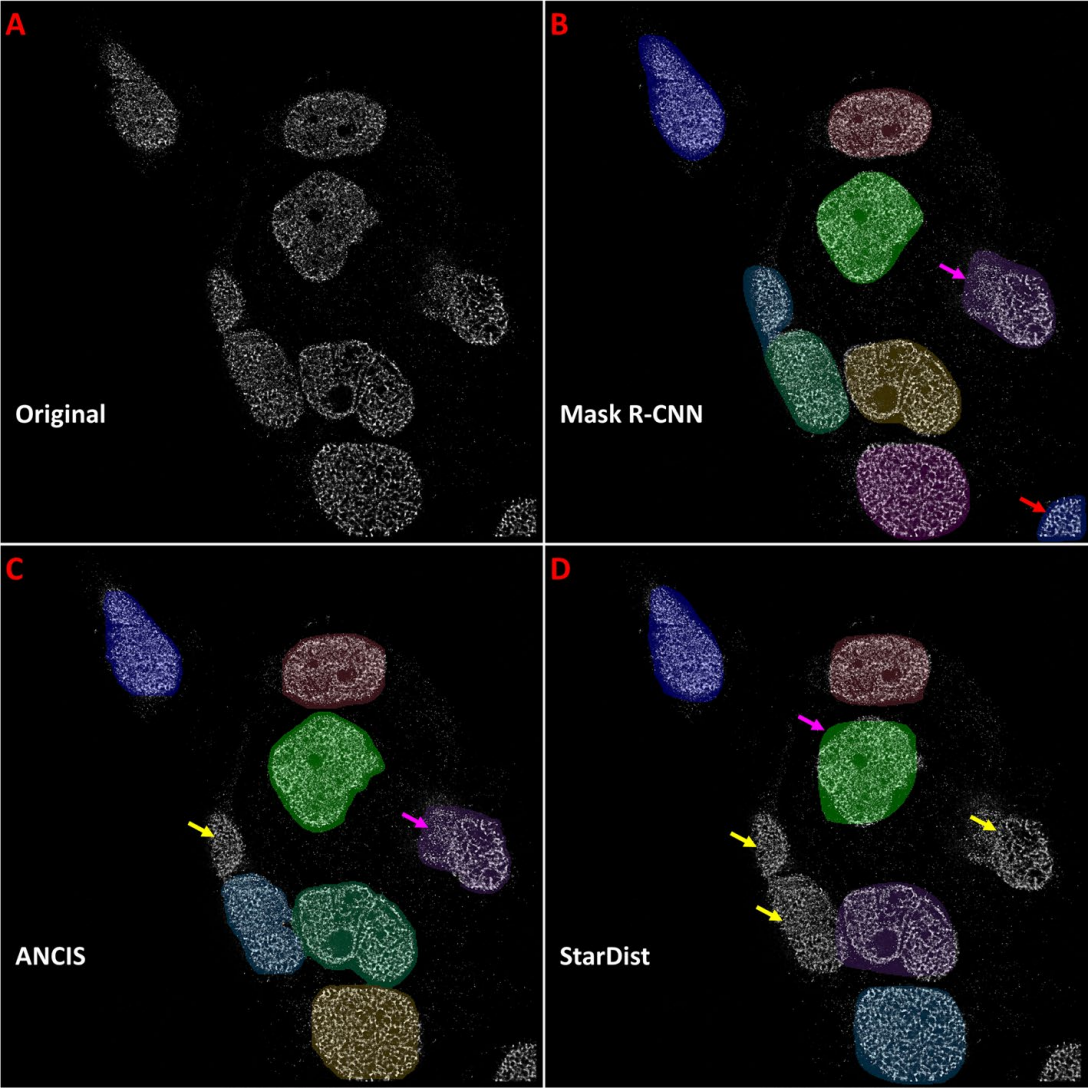
Segmentation examples for STORM image of HK2 cells (H3K4me3). Original test image (**a**) was segmented using (**b**) Mask R-CNN, (**c**) ANCIS and (**d**) StarDist network models. Training and testing were done on cell line dataset B. Red arrows indicate False Positives, yellow arrows point out False Negatives, and pink arrows indicated over-segmentations

Training was initially conducted on cell line dataset A, those images with discrete nuclear texture (e.g., those labeled with RNA polymerase II). Testing of the trained models was conducted on a subset of the STORM images of nuclei also with discrete texture (dataset A), as well as on a set of STORM images of nuclei with dense or diffuse texture (e.g. those labeled with DNA, H3K4me3) (Fig. 5). Nuclei segmentation for cell line images performed higher in test accuracy than the tissue dataset, likely due to reduced cell clustering and more regular cell shapes. Unlike with the tissue dataset, in which Mask R-CNN performed the best overall, top marks for cell line dataset were achieved using ANCIS. When trained using the cell line dataset with dense nuclear texture, all networks achieved F1-Scores above 0.8 (Table 3). Additionally, false negative percent was found to be less than 10% for all trained network models, and Hausdorff distances were also less than 10. Results improved further for ANCIS and StarDist when training was conducted on cell line dataset B, containing cell nuclei with both discrete and dense/diffuse texture. The top results using ANCIS achieved an F1-Score of 0.954, with a false negative percent of 3 and Hausdorff distance of 6.39. Further, networks were also trained and tested on blurred and histogram equalized versions of the images in cell line dataset A. Like with the colon tissue set, the original dataset provided the best results, as neither blurring nor histogram equalization improved the accuracy.

Lastly, we evaluated cross compatibility between cell line and tissue datasets. As mentioned in the previous section, tissue dataset trained network models were applied to segment the cell line imagery. In general, the results were sub-optimal, due largely to under segmentation of the larger cell line nuclei. However, when the cell images were resized down to 256 × 256, the test accuracy improved, particularly for Mask R-CNN (F1 = 0.872) and StarDist (F1 = 0.842). The percent false negatives still remained higher for StarDist, on the downsized dataset, whereas the Mask R-CNN tissue model performed better than its cell line trained models (Table 3). These results led us to contemplate whether a combined training network, incorporating STORM images from both tissue and cell line datasets, could achieve even better performance.

#### Combined dataset

We combined the Kaggle, colon tissue and downsized (256 × 256) cell line dataset B to create a potentially more robust dataset. Downsizing was conducted on the cell line images to roughly match the nuclear sizes to those found in the colon tissue STORM images. Testing on the tissue dataset resulted in improved accuracy over the network trained on the Kaggle dataset alone (Table 1), but worse than the networks trained directly on the STORM images from tissue dataset for both StarDist and Mask R-CNN (Table 3). ANCIS, on the other hand, experienced a boost in the nuclei segmentation accuracy for tissue dataset (F1 = 0.753) compared to the ANCIS model trained on tissue data alone (F1 = 0.739). Since ANCIS had demonstrated a higher false negative percent previously, this boost in accuracy was likely due to an increase in the ability of newly trained model to accept a greater degree of variability in instance identification, learned from the broader dataset with diverse image features. Mask R-CNN, on the other hand, suffered from this same increase in variability, since that network model demonstrated a trend towards over-selection of instances. Test results on the cell line data, however, were surprisingly robust across all networks. Optimal or near optimal F1-Scores, false negatives and Hausdorff distances were found for all three broadly trained network models (Table 3). The Hausdorff distances for StarDist in particular showed significant improvement when trained on the combined dataset, versus when trained on either cell line dataset, (Additional file 6: Figure S6).

### Image processing of test images

#### Noise removal

**Fig. 6:**
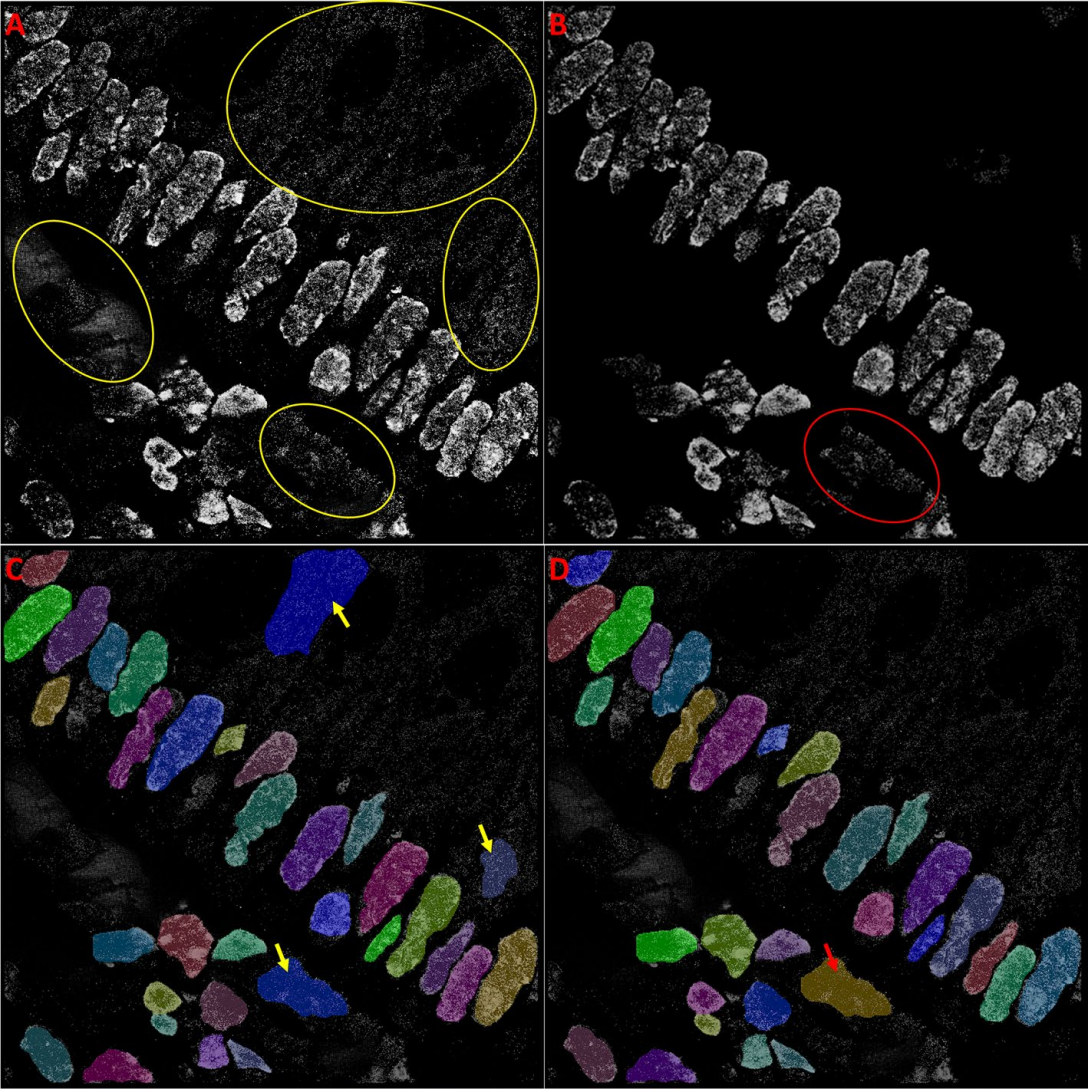
Effect of image denoising on segmentation predictions. A UNet model was trained to identify noise regions (**a**, yellow circles) in our super-resolution images. The identified noisy regions were mapped and subtracted from the original image (**a**) to create a denoised version (**b**), in order to improve test accuracy. When denoising was not performed, noisy regions may be falsely identified as nuclei (**c**, yellow arrows). After denoising, most false positives due to noise disappear (**d**), with the occasional exception of missed noise instances (red arrow in D and red circle in B). Segmentation was conducted using Mask R-CNN

STORM images often contain “noisy regions” due to non-specific binding or unbound fluorophores, out-of-focus fluorescence and autofluorescence signals. Such “noisy” regions are more prominent in the tissue dataset. To further improve accuracy in nuclei segmentation, we conducted noise removal on the test images by training a UNet to semantically recognize and segment noisy regions, as shown in Fig. 6. Test accuracy was slightly improved for all models due to a reduction in false positives. The F1-Scores for Mask R-CNN improved from 0.788 to 0.793, for ANCIS from 0.735 to 0.756 and for StarDist from 0.71 to 0.725, when applied to the tissue test images segmented using tissue trained models. Importantly, false negative percent was markedly reduced, by 5% for both Mask R-CNN and StarDist, and by 12% for ANCIS. The greater improvement for ANCIS, and lesser for Mask R-CNN, is likely due, in part, to the number of total detections. Indeed, noise removal eliminated just as many false positives with Mask R-CNN as with ANCIS. Hausdorff distance was little affected by noise removal. However, it is worth noting that networks trained using more optimal parameters tend to detect less noise, without any additional processing. Using an additional network for noise detection and removal can supplement optimization, but does not replace it.

Application of the UNet trained using tissue dataset noise on the cell line dataset A, resulted in the removal of some nuclei along with the noise, when the nuclei with discrete and sparse texture were erroneously recognized as noise. Therefore, independently trained UNets were required for tissue and cell line images, due to the variation in the noise density between the two different image sets. F1-Scores and false negative percentages were less affected when noise removal was applied to the cell line dataset. On average, for cell line data, the F1-Scores improved by less than 1%, and false negative percent was reduced by less than 2% when segmentation was also conducted by cell line trained network models.

#### Small instance detection and overlap removal

**Fig. 7:**
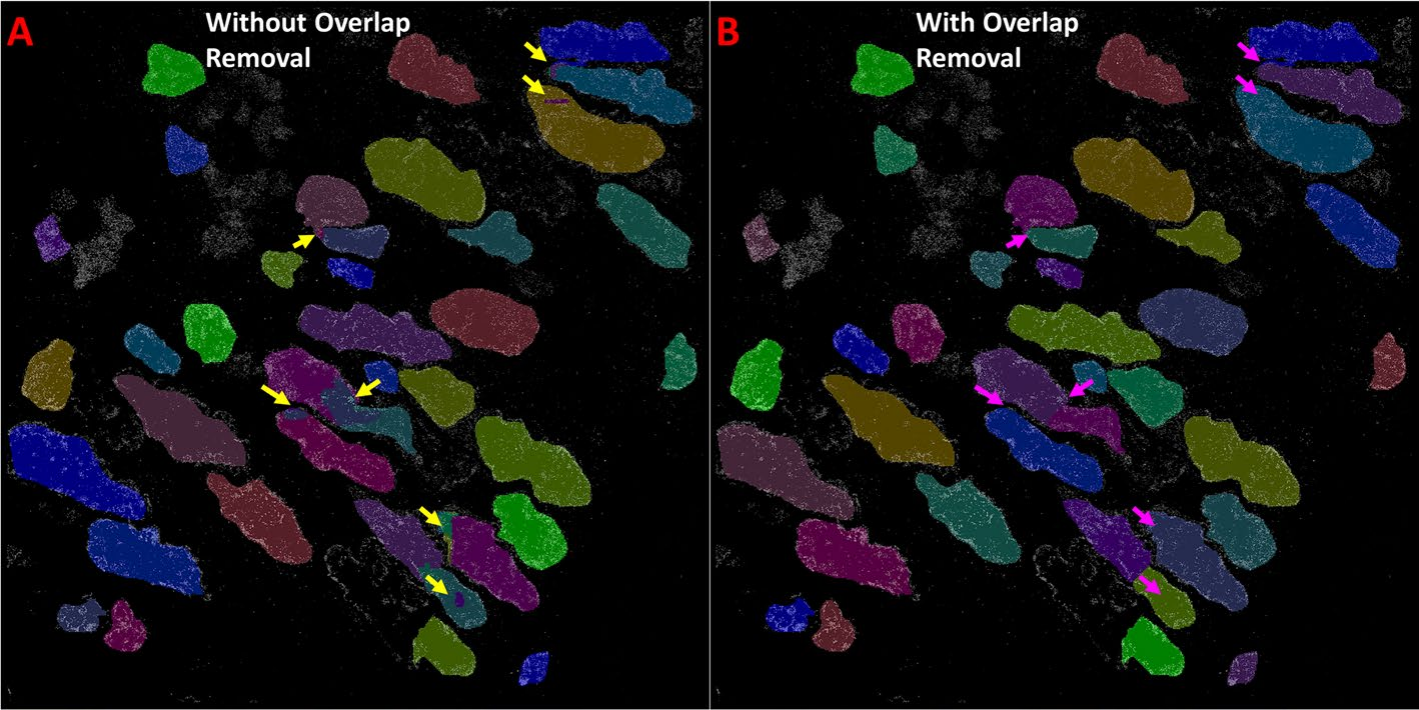
Effect of overlapping instance removal on segmentation predictions. Raw Mask R-CNN-based nuclei segmentations before merging and dividing overlapping instances (**a**, yellow arrows), and after (**b**, pink arrows)

The post-processing steps for the test images, which occurred following instance segmentation, included overlap and small instance removal. These processes made a greater difference in test accuracy with ANCIS and Mask R-CNN (Fig. 7), however StarDist already utilized a built-in module to eliminate overlaps and only benefitted from the small instance removal. The elimination of overlaps and small instance detections improved the F1-Score from the best scoring Mask R-CNN for the colon tissue dataset from 0.774 to 0.793, and the best ANCIS score for tissue data improved from 0.73 to 0.756. The removal of small instances from StarDist tissue segmentation results improved the F1-Score only from 0.716 to 0.725. When applied to cell line dataset A with discrete texture, post processing improved the F1-Score from 0.813 to 0.832 for Mask R-CNN, from 0.883 to 0.902 for ANCIS and from 0.778 to 0.799 for StarDist. Additionally, the false negative percent was reduced by 5 and 17% for Mask R-CNN and ANCIS, respectively, and by 4% for StarDist, when applied to the tissue dataset. False negative percent for the cell line test results only improved by less than 2% for all network models. Interestingly, the Hausdorff distance was not improved by more than 0.25 for any test set.

## Discussion

Super-resolution STORM imaging of nuclear organization has been increasingly used in basic biological research. Recent success in imaging nanoscale chromatin structure on tissue and pathological samples paves the way for its future use in assisting clinical pathology. Nuclei segmentation is the first and essential step towards quantitative analysis of chromatin texture and molecular-scale chromatin folding. However, unlike conventional fluorescence or bright-field microscopic images of cell nuclei, STORM images present several unique characteristics and challenges for nuclei segmentation due to the localization-based single molecule detection. The nucleus often lacks clear borders and uniform texture. As the resolution reaches near molecular scales, many biological structures often appear discrete. In various biological processes or pathological states, the nuclear architecture can also be disrupted. These characteristics make nuclei segmentation very challenging using traditional image processing methods. The success of applying CNNs in nuclei segmentation on various conventional microscopic images motivated us to explore whether it can achieve similar success on the challenging STORM-based super-resolution images.

### Initial testing

The Kaggle dataset included a broad range of stained nuclei images from conventional fluorescence and bright-field microscopy on cells and tissue from different imaging conditions. We found that the Kaggle trained networks severely over-segmented the STORM-based super-resolution images. Notably, our Kaggle trained ANCIS model produced F1-Scores of about 0.01 or less for all test sets. Mask R-CNN, which is more prone to over-selection and under-segmentation, produced the highest test scores, but still failed to segment the images to an acceptable standard. Upon inspection of the segmentation results, we believe the reason for such poor results is the detection of the discontinuous fragments inside the nuclei of super-resolved images as very small instances, largely coming from the inherently discrete or fragmented nuclear texture in the STORM images. Indeed, when we downsized our test set or performed blurring, smaller segments merged into larger detectable pieces, and the test scores improved (although, not by very much). As a result, many of the instances produced were of size less than our minimum test IoU threshold, and so were noted as false negatives. Therefore, training directly on super-resolution imagery was required.

### Network testing

#### Overfitting

We have noted that as our networks were trained with increasing number of epochs, the mean test accuracy, judged by F1-Score, increased up to a point before either decreasing again or leveling off. While increased training typically resulted in decreased noise detection, false positives and overlap, it also created an overfitting condition where the network model failed to recognize valid instances, resulting in increased false negatives. In other words, the network lost some of its ability to infer, and began to discard nominally positive detections. In some cases, we also noticed a drop in test accuracy with increasing dataset size. This condition only occurred with StarDist when trained on the tissue dataset and Mask R-CNN when trained using the cell line dataset. The reason may well again be due to overfitting, although in the case of Mask R-CNN it may have been a fluctuation in test accuracy, as we have seen demonstrated by this network on our dataset. Furthermore, we have found that test accuracy increases rapidly with training set size (Additional file 3: Figure S3). Taken together, these findings indicate that good results can be gained from training sets consisting of tens of images, rather than hundreds or thousands. The exact number will vary depending on image type and target instances. In our own datasets we have observed that fewer cell line nuclei instances than tissue nuclei instances were required to accurately train a CNN for segmentation, due to less variation between instances. In either case, satisfactory results could be attained when training on as few as 20 images.

#### Effects of scale and tiling

Nuclei size often varies significantly (from a few microns to tens of microns) among different cell types, cultured cells and tissues. Scale makes a significant difference in segmentation accuracy [2, 15]. Networks trained on a limited dataset without scale variation or image augmentation, may get locked into recognizing only a limited set of nuclear sizes, resulting in over or under segmentation of instances as well as increased false negatives and splits and merges when applied to a test set containing different nuclear sizes. Many networks are trained to be scale invariant so as to offset these issues by augmenting the training set with scaled versions of the data [3, 11, 17, 21]. Inversely, one study rescaled all of the images so that nuclei were about the same size [15]. Feature Pyramid Networks (FPN) have also been useful in creating prediction feature maps at various image scales [37], and have been used for instance detection with FCNs [38] and with Mask R-CNN [12, 23]. In dealing with the discontinuous image features, typical of STORM-based super-resolution microscopy images, recognizing multiple nuclear sizes may prove particularly important in helping prevent over- or under-segmentation issues.

Additionally, we found that downscaling super-resolution images (i.e. from 5120 × 5120 to 512*512) can be an effective way to handle computational memory limitations involved with processing large scale images. Training and testing on downsized images and then upsizing the segmentation maps to fit the original image scale proved to be a more accurate method than anticipated. The relative positions of the nuclear boundaries in both the downsized images and their labels scaled accurately when upsizing. However, this method was found to be much less effective when scaling ultrahigh resolution images, with each axis containing 10,240 pixels or more. Such large images may be downsized by about 10 folds, however the memory gains are still offset by image size. In these cases, it may be more advantageous to use an image tiling method where each high-resolution image is divided into smaller squares. Network training and testing are then conducted on these squares, and the segmentation results can then be recombined into a full image.

We tested the accuracy of this tiling method (described in Methods) on our colon image dataset, and dividing each 5120 × 5120 image into 512*512 squares, or tiles. Using the same optimal parameters reported for Mask R-CNN in Table 2, we found the accuracy of this tiling method to be comparable to the scaling method reported herein. When training and testing on colon tissue images, the F1-score for the tiling method was found to be 0.783, a drop in accuracy of 0.01 from the scaling method. A slight drop in accuracy could be accounted for by a reduction in network segmentation fidelity to the more detailed nuclear borders found in the full-resolution image tiles. Similarly, when training and testing on cell line images using the tiling method, the test accuracy dropped from 0.831 to 0.818.

#### Performance of nuclei segmentation using different networks

Under the same settings, including epochs, steps, RPN and confidence threshold values as well as maximum instance limits, ANCIS models demonstrated increased false negatives whereas Mask R-CNN models had more false positives when trained and tested on the same dataset. Two distinct differences may have given rise to this condition, the first being the different region proposal networks used (SSD versus Faster R-CNN) and the second being the different segmentation network (UNet versus Mask R-CNN). One item of note in favor of identifying the segmentation network as the culprit is that the greater number of false negatives occurred when applying the UNet based StarDist network as well; this suggests UNet may be a more selective algorithm than Mask R-CNN. An additional difference between the networks is the extra modules. StarDist incorporates a module for eliminating overlaps, while ANCIS incorporates a module for distinguishing intricate object features from background. These modules, however, perform more as supplements to improve segmentation border accuracy, and should not cause or prevent over or under selection of instances.

It is worth noting that the quality of nuclei segmentation may not be fully reflected by the quantitative metrics on the test results (e.g., F1-score) for each network. The overall qualitative perception on the segmented nuclei can also be an important factor to consider. Comparing network performance, certain general features became apparent. Mask R-CNN appeared to achieve the greatest number of detections, in both true positive nuclei and false positives consisting of mainly noise and small, off-target nuclei (Figs. 4B & 5B, Additional file 4: Figure S4). Both Mask R-CNN and ANCIS utilized region-based localization, which resulted in some overlapping detections. ANCIS, however, appeared to detect less noise than Mask R-CNN, a quality reflected in the increased false negative percent found in the data. StarDist generally detected less noise as well, but also made fewer true positive detections, again indicating that UNet is a stricter segmentation algorithm than Mask R-CNN. The Hausdorff distances were lower for Mask R-CNN and ANCIS than for StarDist, possibly due more to the star-distributed polygon fitting module creating a more rounded border, rather than the segmentation network.

In addition to training each network on cell line image sets with discrete nuclear texture, we also trained on a broader cell line dataset containing various labeled molecular targets with both discrete and dense or diffuse nuclear texture. This introduced greater variability to the training set, potentially making it more robust. The broadly trained cell line network models demonstrated increased test accuracy for both ANCIS and StarDist networks. The Mask R-CNN model, however, did not perform as well using our broader training set. In fact, the increased variability appearing to cause increased splits and merges of instances. One reason may be that the increased variability in the training set confused a segmentation network that is already more prone towards over selection. We have seen that ANCIS and StarDist, both of which are built on UNet, and both of which demonstrated greater false negatives than Mask R-CNN, can enjoy reduced false negatives when trained on a broader dataset. Mask R-CNN on the other hand appeared to perform better when trained more specifically.

Training segmentation networks on one image type, such as from the cell line dataset, and testing on another, such as our colon tissue test set, typically produced poor results. Even when both training and testing on either tissue or cell line images, the results may suffer if the test set presented a dramatically different texture or image features than the training set. Such a result was observed when training on our colon tissue data and testing on the prostate tissue images, where the F1 segmentation accuracy dropped between 5 and 15% points compared to the results found when segmenting the colon tissue test set. In most cases, while a pre-trained network can be a good starting place, and worth trying out, it is better to train the network on the same type of image that is being segmented.

### Post processing

#### Noise detection

Well-trained networks are more robust against noise, and therefore do not require as much post-processing, however noise removal can still be beneficial as long as it is not too aggressive. When training a noise detection network, care must be taken to train on noise similar to that found in the test set to avoid misclassification issues. For example, if only very dense noise is used to train a noise removal network for a dataset containing nuclei with various textures and label densities, including low density, discrete or diffuse labelled nuclei, then some of those may be misidentified as noise and removed (Additional file 7: Figure S7). Noise removal is especially effective on STORM images in the tissue dataset, where large “noisy” regions can be prominent due to the out-of-focus fluorophores and autofluorescence. Therefore, it is typically helpful to train a noise recognition network on the same, or similar, training set as used for instance detection.

#### Overlap removal

The method used for merging or, more particularly, splitting overlapping instances may not be ideal. However, we found that for nuclei segmentation on STORM images, a significant improvement in test accuracy was gained even by roughly splitting, or merging, the overlapping cells. An imprecise solution, as demonstrated in the methods section (Fig. 10), can still improve test accuracy by as much as 5–10% points on the F1-Score. Methods that more accurately divide the overlap will not add more than 1–2 additional percentage points, in most of the cases we have observed. While this gain is nonetheless desirable, no method will always achieve perfect results.

Overall, despite the small gain on test accuracy metrics due to noise removal and post processing, we believe it is worth attempting. Even with post processing, there will still be segmentation errors. Those errors can, however, and at the user’s discretion, be manually corrected. While this is a time-consuming endeavor, it is certainly less so than segmenting all of the instances manually.

## Conclusion

In conclusion, we evaluated the performance of a set of three CNNs for nuclei segmentation on STORM-based super-resolution fluorescence images from a diverse dataset from cell lines and tissues with different labeled molecular targets at different biological and pathological states. We found that Mask R-CNN has the best overall performance for nuclei segmentation of STORM-based super-resolution images, when trained on the STORM images from tissue alone, with high test accuracy, low false negatives and good border identification (short Hausdorff distance). The pre-trained model on the tissue dataset can achieve a good performance on the downsized images from cell lines as well. Optimal performance will still be achieved when training and testing are conducted on the same data type. Image processing such as noise removal and overlap removal helped improve the overall accuracy, especially on the tissue dataset. We have built our software using the Python platform, and used GitHub and Google Colaboratory to disseminate to the biomedical research community (https://github.com/YangLiuLab/Super-Resolution-Nuclei-Segmentation).

## Methods

### Data collection

Images were collected using our custom-built STORM system on both formalin-fixed, paraffin-embedded (FFPE) tissue section and cultured cells. The imaging systems, sample preparation, image acquisition and reconstruction have been previously described [5, 7]. The image characteristics of cell nuclei can vary dramatically depending on the labeled targets. We included a diverse set of STORM images of nuclei with different molecular targets that exhibit various distinct nuclear textures (e.g., discrete clusters, diffuse pattern, or dense clumps) from the various labeled molecular targets from different biological or pathological states. Additional file 8: Table S1 shows the list of cell/tissue types, labeled target in the nuclei, biological or pathological states of the cells or tissue and fluorophore used.

All STORM-based super-resolution images were originally captured at 5120 × 5120 pixels, and were resized for speed during training. Downsizing was conducted using bilinear interpolation with averaging. Datasets were constructed by creating 1024 × 1024, 512 × 512 and 256 × 256 versions of the STORM images of cell nuclei from human colon tissue as well as 512 × 512 and 256 × 256 versions of the STORM images of cell nuclei from cell lines.

**Fig. 8:**
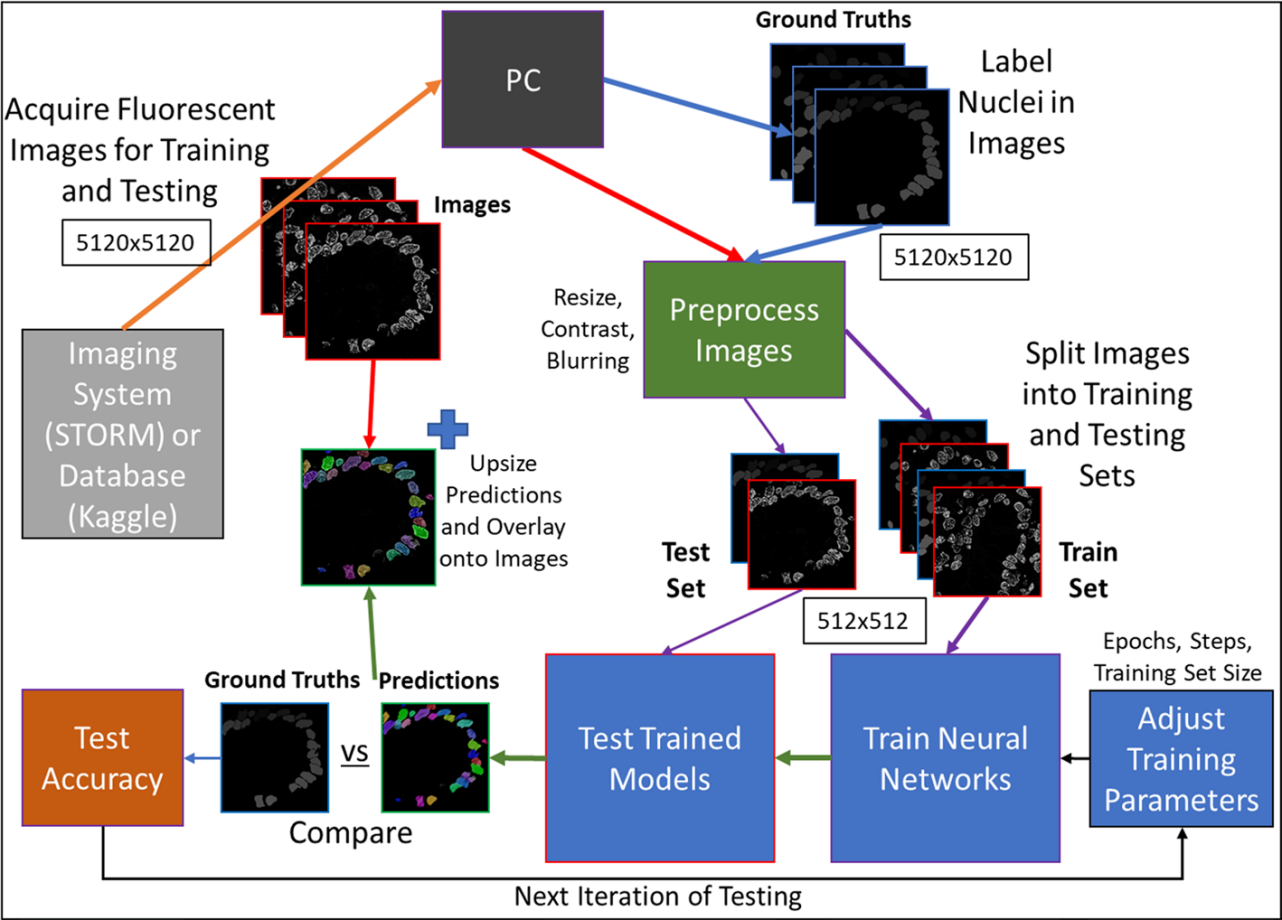
Workflow for STORM image acquisition, labeling, processing, network training and testing. Sequence begins at the top left and proceeds clockwise

Additional datasets were created for training and testing by blurring or contrast enhancing the STORM images of nuclei from human colon tissue and cell lines with resized 512 × 512 and 256 × 256 datasets. Blurring was applied using a Gaussian filter with sigma set at 2. Contrast enhancement was conducted by means of histogram equalization with normalization, to limit intra image variations. All image resizing and pre-processing steps described in this section were conducted using Fiji [39]. Our workflow is depicted in Fig. 8.

#### STORM images of cell nuclei from tissue samples

A total of 134 STORM images of nuclei labeled with heterochromatin marker (H3K9me3) were included in the human colon tissue dataset, of which 60% were used for training, 30% for testing and 10% for validation. The same testing, training and validation images were used for each network. Additionally, a set of 69 STORM images of nuclei stained with the same marker from mouse prostate tissue (from Myc-driven prostate tumorigenesis mouse model and wild-type mice) were used as an alternate test set for the network models trained on colon tissue data. These datasets include normal tissue and those at different pathological states (low-grade and high-grade precancerous lesions and invasive cancer). As we have previously shown, chromatin compaction becomes progressively more disrupted in carcinogenesis and therefore chromatin texture varies significantly in normal tissue, precancerous lesions, and invasive cancer [7].

#### STORM images of cell nuclei from cell lines

Two training sets were collected for the cell line dataset. Cell lines used in this dataset include mouse and human fibroblast cells, human breast cancer cells, kidney cells and prostate cancer cells. A wide variety of molecular targets in the cell nuclei (H3K27me3, H3K4me3, DNA, RNA polymerase II) were labeled, which are characteristic of a diverse “texture” of nuclear organization. These datasets were generally divided into two categories based on its image characteristics: discrete and dense nuclear texture, which depend on either molecular targets or biological states. A total of 44 STORM images with discrete nuclear texture and an additional 65 images with dense nuclear texture were used in this study. Each dataset was divided: 60% for training, 30% for testing and 10% for validation. The STORM images of cell nuclei with discrete nuclear texture comprised our first, single-source (single labeled target of RNA polymerase II) cell line training and validation dataset (Additional file 9: Figure S8), herein referred to as *cell line dataset A*. Next, the STORM image training and validation datasets including images with both discrete and dense nuclear texture comprised our second, multiple-source (i.e., various labeled targets) training and validation set, which we shall call *cell line dataset B*. Then, the two test sets from cell line datasets A & B were combined to create a single test dataset for both the discrete and dense nuclear texture cell line training sets. In this way, we directly compared the network performances of both models: trained on a single data source presenting a discrete texture, versus trained on multiple sources containing both discrete and dense texture. A more complete breakdown of cell type, biological state, labeled molecular target and fluorophore used in each image set, as well as a breakdown of which dataset each image type appears in (Additional file 8: Table S1).

#### Kaggle dataset

In addition to our own STORM super-resolution image dataset, we also trained and tested our selection of neural networks using the 2018 Kaggle data science bowl dataset [1]. Included in this publicly available nuclear image dataset were wide-field fluorescence, H&E stained and brightfield microscopic images of nuclei from cultured cells and tissue. From the entirety of the dataset, we utilized only the stage 1 training set, containing 670 labeled images. Kaggle datasets were not resized, but rather tested as is.

### Neural network implementation

All networks were implemented using Python 3.6.9 with Tensorflow 1.14 and Keras 2.3.1. Training and testing was conducted on an Alienware X51 r3 PC running Ubuntu 18.04 LTE, implementing an Intel® Core™ i7-6700 (8 MB Cache, 4.0 GHz) CPU with 32 GB (2133 MHz) DDR4 RAM and a NVIDIA GeForce GTX 1060 GPU with 6 GB memory.

All networks utilized the Adam optimization algorithm to iteratively update network weights. Additionally, all networks used image augmentation to increase the robustness of the training datasets. Augmentation methods included random horizontal and vertical flipping, rotations of 90 or 270 degrees and scaling by values between 0.5 and 1.5. Non-linear transforms such as stretching, skewing or sheering were not used.

### Software distribution

To make our methods more readily available and duplicable, we have coded a set of Colab notebooks on which users can both test and train the neural networks used in this study (https://github.com/YangLiuLab/Super-Resolution-Nuclei-Segmentation). Colab notebooks are designed to run using a hosted runtime on the Google cloud service. This allows users access to all of the necessary coding libraries, as well as GPU compute power, without having to install and setup the environment locally. Notebooks were created for Mask R-CNN and ANCIS, as well as the UNet used herein for noise detection. A separate notebook for StarDist was not created, since a good Colab notebook focusing on nucleus segmentation already exists for this network, and can be used with our pretrained weights [40]. Our modifications for post processing predictions and resizing images were also included in the code, as optional parameters. We have also included links to our pre-trained weights for each network using our super-resolution image sets.

#### StarDist

The StarDist distribution applied here was version 0.6.0, downloaded from the author’s GitHub page [20]. Parameters used included a batch size of 2 and a training patch size of 128 × 128. The number of rays for the star distributed polygons was set at 32, the author recommended value. During prediction, the non-maximum suppression (nms) threshold was set to 0.3 and the probability threshold to 0.5. Additional settings were left to their default values, including binary cross-entropy training loss function and mean average error polygon distance loss function parameters.

#### ANCIS

Our tested distribution of the ANCIS code was also downloaded from the author’s GitHub page [14]. Network parameters used for all training and testing included a nms threshold of 0.7, confidence threshold of 0.5 and segmentation threshold of 0.5. Thresholds were selected based on previous experiences with image segmentation networks, as well as findings reported in the literature. The batch size was limited to 2 by the GPU capacity. The maximum detectable instance limit was set greater than the number of instances in any of the training or test images, we used 400, since we did not wish to limit segmentation in this way. Anchors were left at defaults values, along with default cross entropy loss function.

#### Mask R-CNN

Matterport’s broadly disseminated Mask R-CNN distribution, version 2.1, was implemented in these experiments [41]. Threshold parameters and maximum instance limits were matched to those reported above for ANCIS. In addition, we applied some of the parameters set by Waleed Abdulla in his nucleus segmentation example code [41]. Anchors per image were set to 64, and anchor scales were set at (8, 16, 32, 64, 128). We did not implement mini masks, however, and our batch size was limited to 1. The network backbone used was resnet50, and binary cross entropy loss function as used. Training was initialized on the pre-trained coco dataset weights, downloaded from the code author’s website [41].

### Network training and testing

#### Initial testing

Each of our tested neural networks came packaged with a pre-trained model using the Kaggle stage 1 dataset. We evaluated the performance of these broadly trained nuclei detection models on our super-resolution images by calculating the F1-Score of the segmentation results. Images used for segmentation included our STORM images of nuclei from human colon tissue and cell line test sets (original 5120 × 5120 image size downsized to 512 × 512). The F1-Scores were calculated at an IoU threshold of 0.7, using Caicedo’s method [21]. Additionally, we tested the effect of resizing by testing the Kaggle models on the same datasets downsized to 256 × 256. To evaluate whether converting the super-resolution images into their lower-resolution versions improve the performance of nuclei segmentation, we also tested the models on blurred and histogram equalized versions of our 256 × 256 test sets.

#### Optimization

To identify the optimal parameters, we performed training and testing over a range of variable parameters including epochs, steps per epoch and learning rate. Performance comparisons were conducted by tabulating the F1-Scores on the resultant test data, evaluated at an IoU of 0.7, using the method set down by Caicedo et al. [21]. Optimization was conducted for the colon tissue dataset and also for the cell line A dataset (discrete nuclear texture). First, a model for each network was trained using 5, 10, 20, 50, 100, 200, 400, 600, 800 and 1000 epochs, then each epoch’s model was tested for comparison. StarDist and Mask R-CNN networks were then trained using the optimal number of epochs with 20, 50, 100, 200, 300, 400 and 500 steps, and again tested. ANCIS was not evaluated for number of steps as the code did not provide the option to change steps, however ANCIS did provide separate training functions for both the region proposal and segmentation networks, and both were optimized for epochs. Lastly, each network was trained using learning rates of 1e−3, 1e−4 and 1e−5.

In order to determine the minimum number of required images in the dataset to achieve acceptable results, we varied the size of each dataset used for training. Using the tissue dataset, the training set size was varied from 10, 20, 40, 60 and 77 images; for the cell line dataset, we used 10, 20, 40 and 65 images. Models were retrained for each training set size, and accuracy testing was conducted for each network.

#### Network evaluation

Using the optimal settings determined in the previous section, we conducted a more thorough testing and evaluation of each network’s performance on our STORM images. Test accuracy was analyzed using the F1-Score, Hausdorff distance and the false negative percentage, again using an IoU threshold of 0.7. The Hausdorff distance was calculated using the scikit-image version 0.17.2 python library. Both the Hausdorff distance and the F1-Score were averaged across all instances that achieved the requisite IoU threshold in each test dataset. The false negative percentage was determined as the total number of ground truth instances that did not achieve the requisite IoU score in the predictions of each test set divided by the total number of ground truth instances in that set. Similarly, the false positive percentage was calculated as the total number of predicted instances without a corresponding ground truth instance, divided by the total number of ground truth instances in the image.

The optimally trained colon tissue network models were used to evaluate both the colon tissue and prostate tissue test sets, as well as the 512 × 512 and 256 × 256 cell line test data. Additionally, the trained network models for cell line training sets A & B were both used to evaluate the cell line test set images. Only the network models for cell line training set B (containing both discrete and dense nuclear texture) was applied to the 512 × 512 and 1024 × 1024 colon tissue test sets. Both the colon tissue and cell line network models were used to create two new datasets (each) by either blurring or performing a histogram equalization on the images. Networks were trained on the blurred or equalized images using the same optimal parameters as the original image datasets were trained on, and then tested on their corresponding blurred or equalized test sets.

### Segmentation processing

#### Noise removal

**Fig. 9:**
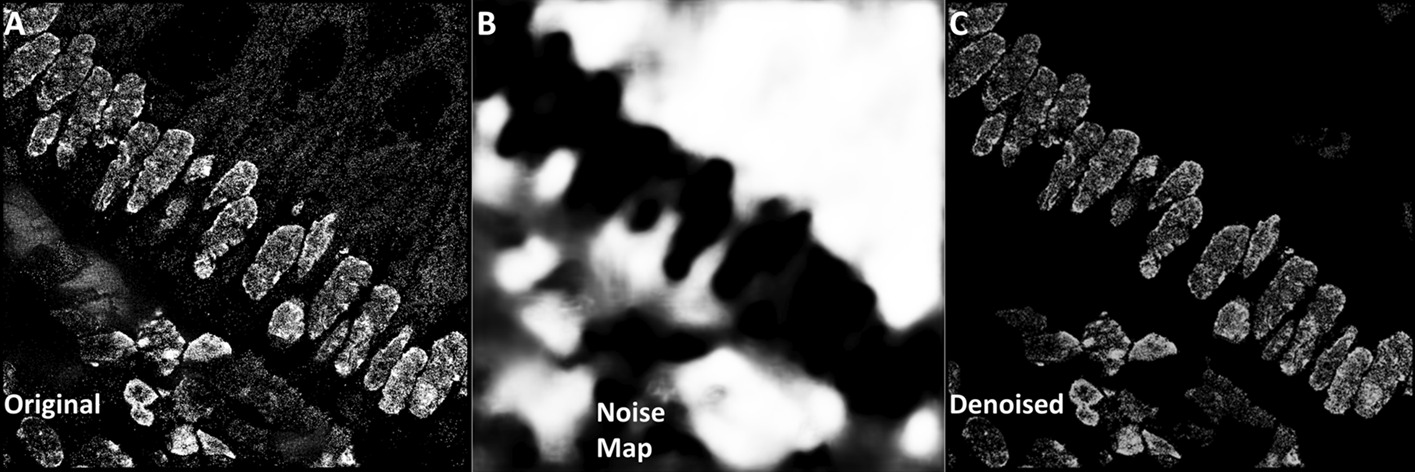
Denoising super-resolution images using UNet. Noisy super-resolution image from our colon tissue dataset (**a**), noise probability map (**b**) output from a UNet model trained on similar noise regions from super-resolution images, and the denoised image (**c**), resulting from subtracting the noise map from the original image in (**a**)

Test images were pre-processed before segmentation. A semantic UNet [11] was independently trained on the human colon tissue and cell line B training sets, using labels indicating not nuclei, but rather noise regions, consisting of out-of-focus nuclei or unbound fluorophores. The UNet was trained for 50 epochs at 300 steps per epoch, with a learning rate of 1e−4. Image augmentation, like that used for training our instance detection networks, was applied, along with the Adam optimizer (0.9 momentum) and binary cross entropy loss function. The trained models were then tested on their respective test sets, creating noise probability maps as the outputs (Fig. 9). The probability maps were binarized using Otsu’s method and subtracted from their respective test images, creating our denoised test sets.

#### Post processing

**Fig. 10:**
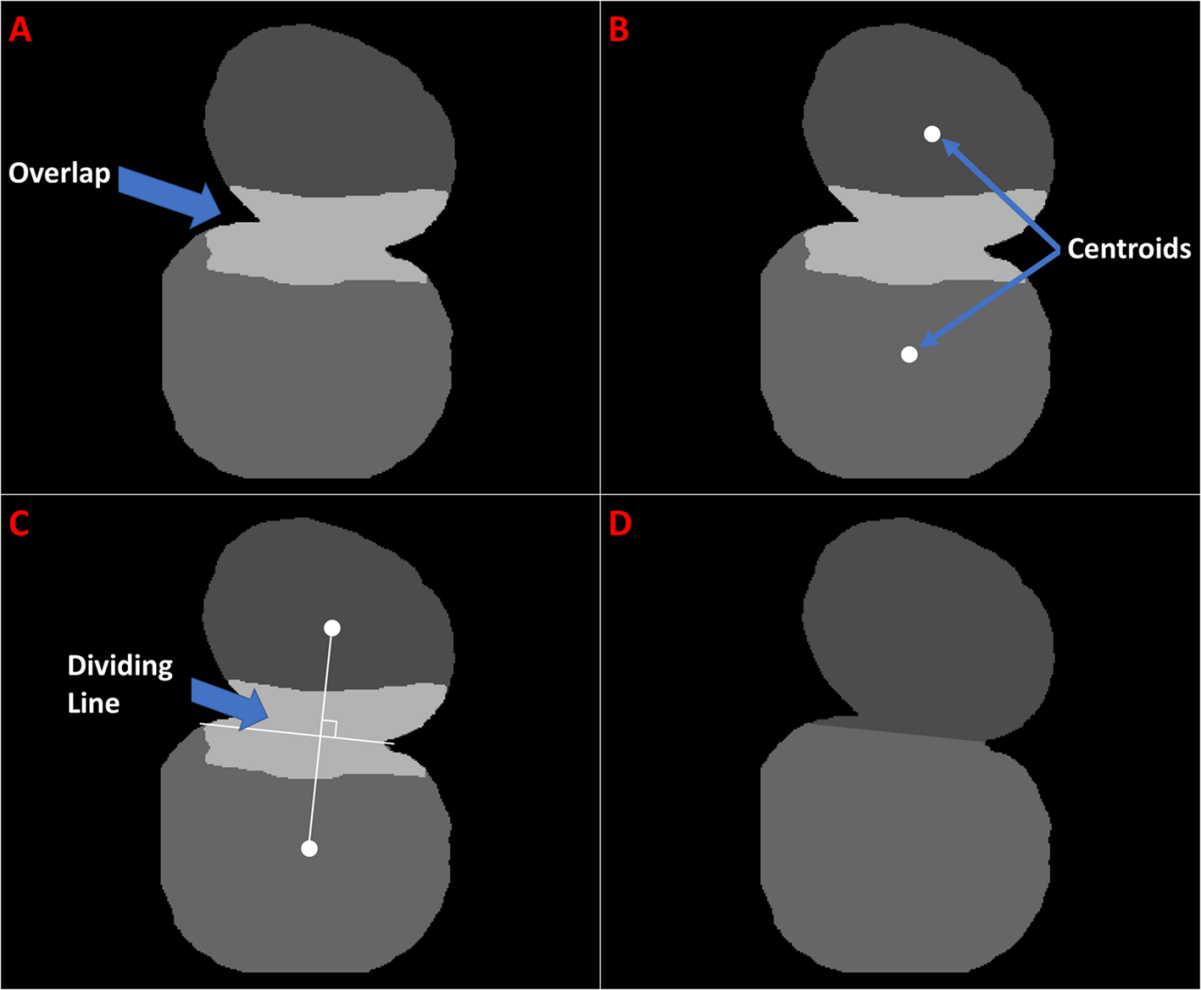
Method for splitting merged instances. (**a**) The lighter area is where the two instances overlap, (**b**) The center of each instance is identified, (**c**) Centers are connected, and the dividing line is drawn perpendicular to the connecting, and through its center point, (**d**) Instances are relabeled without overlap

Following test image segmentation, post processing was applied. First, small instances with pixel area of less than 25% of the image average, were removed. Next, overlapping instances were merged or separated. Where overlapping instances were located, the pixel area of each instance was calculated as was the area of the overlap. Instances sharing more than 50% of their total pixel area, or where 50% of either instance’s pixels overlap, those instances were merged. When the overlapping area comprised less than 10% of one instance, but more than 10% of the other, the overlap was assigned to the instance with the greatest overlap. However, when both instances contributed 10% or less of their pixels to the overlapping region, the entire region was randomly assigned. Lastly, when the overlap comprised less than 50% of each instance, but more than 10%, the overlapping region was split, as shown in Fig. 10.

The first step in dividing an overlap was to find the center of mass of each instance. Next, the two center points were connected by a line segment. The center of the line was then found, and a new line was created, perpendicular to the first and passing through its center point, as well as through the borders of the overlapping regions. The overlapping instances were then split along the second line and each section assigned a different label. Any new small instances created during the split were again removed.

#### Tiling

**Fig. 11:**
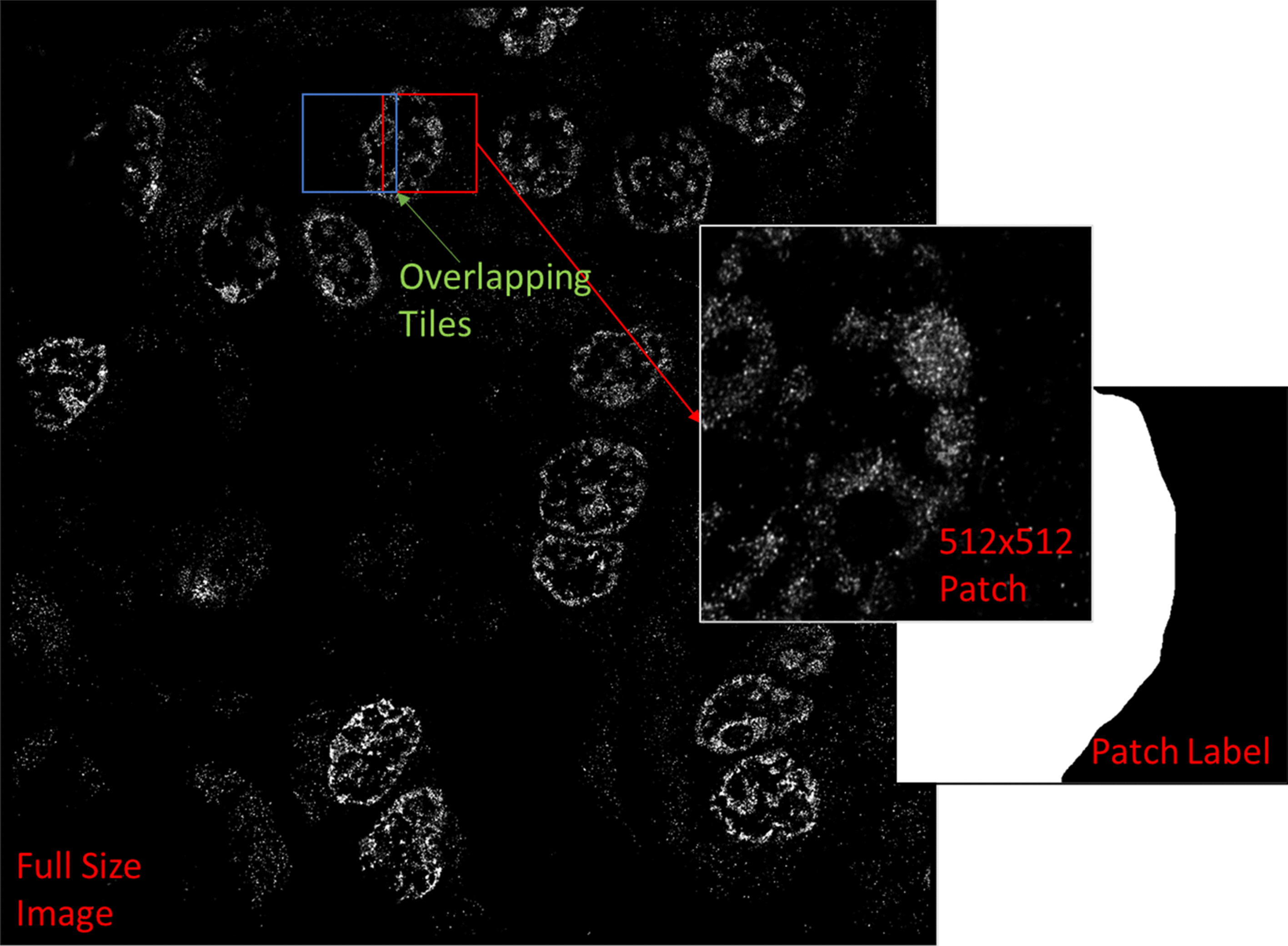
Example of dividing large image into square tiles. A large 5120 × 5120 super resolution image is divided into many smaller image patches, or tiles. The image label, identifying fluorescent nuclei, is similarly divided. A tile overlap is used for test images, to be segmented by a trained network, in order to help recombine overlapping predictions and remove tile borders

Training neural networks directly on large format, super-resolution images can be a slow and memory intensive process. To offset memory limitations of our system, we downsized our images prior to training. Segmentation results from a trained network were later upsized to match the original image. We compared the accuracy of our method to a tiling method, where each image in the training and testing datasets was divided into a sequence of smaller images. These images, which would retain the detail of the original super-resolution images, were then used to train the networks along with their corresponding labels (Fig. 11).

When conducting segmentation on test images, the original images (5120 × 5120) were divided into (512 × 512) squares with each square overlapping its neighbors by a 128-pixel margin. The purpose of the overlap is to help merge predictions when reuniting the segmented image squares. This is accomplished by sampling and matching the labels of overlapping segments, eliminating false segment divisions created by tile image borders (Additional file 10: Figure S9). Resulting whole test image segmentations were analyzed for accuracy.

## Supplementary Information

**Additional file 1: Figure S1.** Mask R-CNN trained on the Kaggle dataset and applied to STORM images. Using a Kaggle data trained Mask R-CNN, examples of the best results we can achieve when conducting nuclei segmentation on super-resolution images from cell lines and tissue with distinct nuclear texture. Downsizing the test images (A-D) and applying the neural network to densely labeled cell line nuclei (A) can result in very good segmentation. When applied to less dense nuclei with discrete (B) or diffuse (C) structures, over or under segmentation can occur. Additionally, the trained network can perform well on tissue images where the nuclei exhibit bright intensity and are well separated (D). However, the results here, with the exception of (A), are not as good as those found when applying Mask R-CNN trained directly on STORM data.

**Additional file 2: Figure S2.** Training steps per epoch and RPN versus test accuracy. (A) Training steps per epoch versus test accuracy for Mask R-CNN and StarDist trained on the STORM images of human colon tissue dataset. Results for the cell line datasets proceeded similarly, only at higher F1-Scores. After early fluctuation, the accuracy continued to grow with steps for Mask R-CNN up to 500 steps. StarDist test accuracy, however, slowly rose to a peak at 300 steps before falling off. ANCIS did not provide a step-per-epoch variable, but provided separate training programs for its region proposal network (RPN) and segmentation algorithm. RPN epochs versus test accuracy plot (B) for ANCIS rose quickly between 0 and 50 epochs, then more slowly until reaching a peak at 600 epochs.

**Additional file 3: Figure S3.** Test accuracy versus number of images in the training set. Training set size had a distinct effect on test accuracy when conducting training for each of the three networks on the colon tissue dataset (A) and the cell line A dataset (discrete nuclear texture) (B). All networks demonstrate a general improvement in accuracy with number of images, with exceptions. In the tissue set, the Mask R-CNN test accuracy fluctuated, although the largest training set still provided the best accuracy. When training the cell line dataset, it was StarDist that fluctuated, while Mask R-CNN performed best when trained on 60 images rather than the maximum number.

**Additional file 4: Figure S4.** Example Mask R-CNN segmented STORM images of colon tissue at normal and various pathological states. States include (A) normal healthy tissue, (B) a low-grade dysplasia, (C) a high-grade dysplasia, and (D) an invasive adenocarcinoma. Results for the normal state were the most accurate, followed by low-grade, high-grade and invasive cancer. This demonstrates a decrease in test accuracy with decreasing nuclear cohesion. Segmentation was conducted using Mask R-CNN trained on the STORM colon tissue dataset.

**Additional file 5: Figure S5.** Nuclei segmentation on STORM images from prostate tissue dataset. Segmentation of prostate tissue images using models which were pre-trained on the STORM colon tissue dataset. The original STORM image of prostate tissue (A) was segmented using (B) Mask R-CNN, (C) ANCIS and (D) StarDist networks. All three networks had multiple false negatives, however border placement appeared fairly accurate on the true positives. Mask R-CNN performed the best on this tissue dataset.

**Additional file 6: Figure S6.** Improved nuclei segmentation of StarDist method when trained on a mixed dataset. Representative nuclei segmentation of the StarDist algorithm on our cell line test images when training was conducted on our cell line A dataset (A), versus when trained on a combined dataset (B) (dataset included Kaggle, STORM colon tissue, and STORM cell line images). Similarly, we trained StarDist on our colon tissue dataset and applied the model to our colon tissue test set (C), and then applied the StarDist trained on the combined dataset to the same test set (D). Training on the combined dataset clearly improved instance border accuracy, particularly for the cell line images (B), and reduced false negatives in the tissue images (D). ANCIS demonstrated a similar improvement when trained on the combined dataset and tested on the tissue images. Mask R-CNN alone did not improve when trained on the combined dataset.

**Additional file 7: Figure S7.** Improper noise model can result in nuclei deletion when denoising. When conducting denoising using an improperly trained UNet noise model (trained only on dense noise), less noisy super-resolution images from our colon tissue dataset (A), which also contain less densely labeled nuclei, sometimes suffered from nuclei deletion (C) due to the networks inability to accurately distinguish noise from cell data (B). The noise probability map (B) from the UNet model shows a high probability of suspected noise (white regions) over several nuclei sites. The resulting denoised image, when this model is applied to the original image (A), shows the deletion of real nuclei (C).

**Additional file 8: Table S1.** Cell types included in our STORM datasets, along with their respective labeled targets, biological states, fluorophores and datasets.

**Additional file 9: Figure S8.** Example cell line images, each expressing different textures. Textures include a dim, diffuse pattern (active form of RNA polymerase II in 3T3 cells) (A), more densely placed labels (DNA in 3T3 cells) (B) and (H3K4me3 in HK2 cells) (C), and discrete labels (H3K27me3 in CA1h cells) (D).

**Additional file 10: Figure S9.** Tiling method example with and without overlap. When the test image is divided into non-overlapping squares, or tiles, for segmentation, the borders between tiles will act as artificial borders between labeled detected instances upon image reconstruction, creating divided predictions (upper diagram). Overlapping allows for a post-segmentation program to sample the overlapping region in adjacent tiles and merge overlapping instances during reconstruction (lower diagram).

## Data Availability

Raw images used for this study are not publicly available, but can be made available upon request, due to the large size of the raw data. Representative images as well as the code used in this study have been made available on the authors GitHub (https://github.com/YangLiuLab/Super-Resolution-Nuclei-Segmentation). 2018 Data Science Bowl data can be found on the Kaggle website: https://www.kaggle.com/c/data-science-bowl-2018/data.
