# Supplementary material for: Application of convolutional neural networks towards nuclei segmentation in localization-based super-resolution fluorescence microscopy images
Source: BMC Bioinformatics. 2021 Jun 15;22:325. doi: 10.1186/s12859-021-04245-x (PMC8204587; doi:10.1186/s12859-021-04245-x)
Supplement: Supplementary file 1 — Additional file 1: Figure S1. Mask R-CNN trained on the Kaggle dataset and applied to STORM images. Using a Kaggle data trained Mask R-CNN, examples of the best results we can achieve when conducting nuclei segmentation on super-resolution images from cell lines and tissue with distinct nuclear texture. Downsizing the test images (A-D) and applying the neural network to densely labeled cell line nuclei (A) can result in very good segmentation. When applied to less dense nuclei with discrete (B) or diffuse (C) structures, over or under segmentation can occur. Additionally, the trained network can perform well on tissue images where the nuclei exhibit bright intensity and are well separated (D). However, the results here, with the exception of (A), are not as good as those found when applying Mask R-CNN trained directly on STORM data. [file 12859_2021_4245_MOESM1_ESM.pptx]

## Slide 1
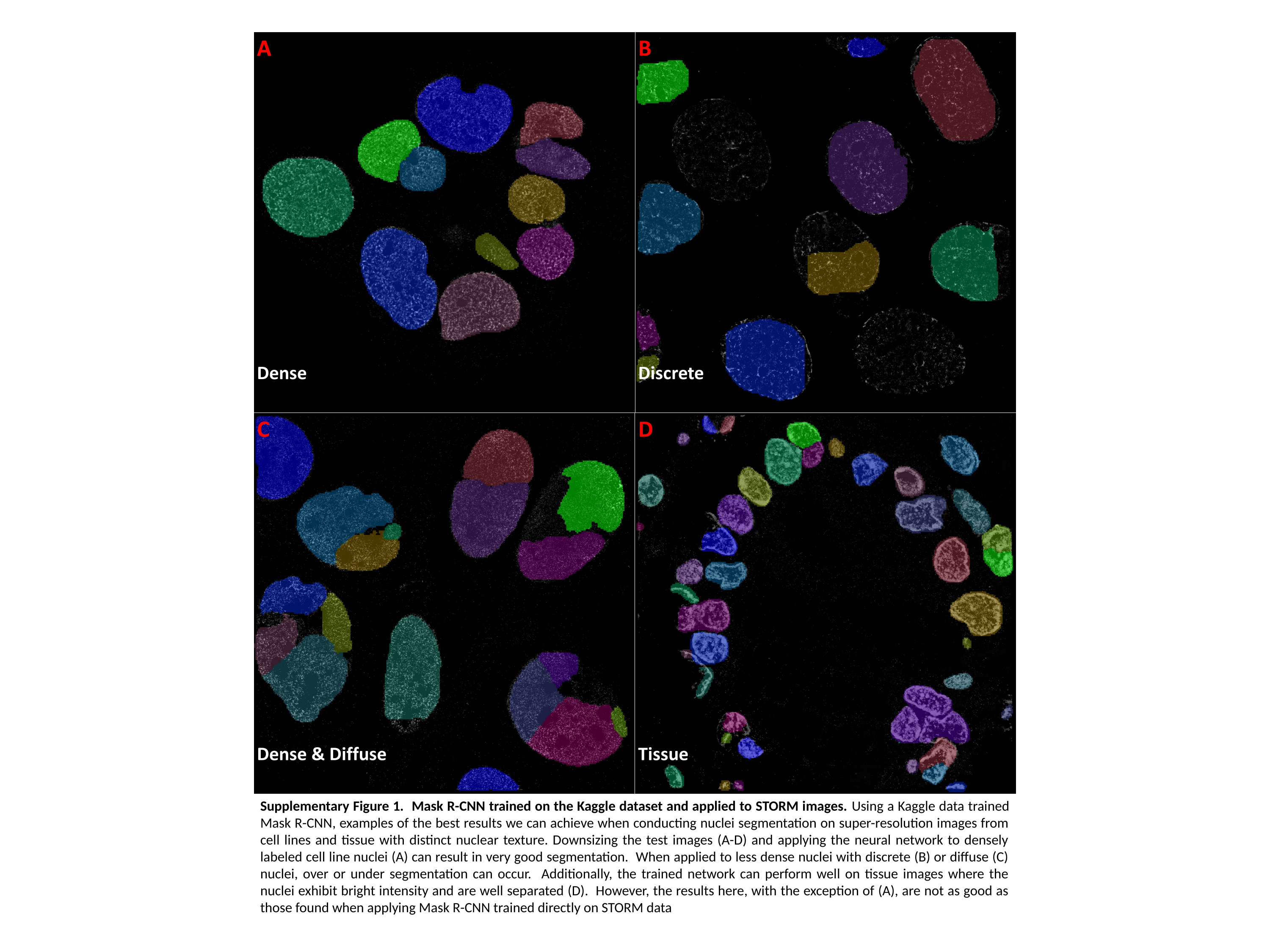

Supplementary Figure 1. Mask R-CNN trained on the Kaggle dataset and applied to STORM images. Using a Kaggle data trained Mask R-CNN, examples of the best results we can achieve when conducting nuclei segmentation on super-resolution images from cell lines and tissue with distinct nuclear texture. Downsizing the test images (A-D) and applying the neural network to densely labeled cell line nuclei (A) can result in very good segmentation. When applied to less dense nuclei with discrete (B) or diffuse (C) nuclei, over or under segmentation can occur. Additionally, the trained network can perform well on tissue images where the nuclei exhibit bright intensity and are well separated (D). However, the results here, with the exception of (A), are not as good as those found when applying Mask R-CNN trained directly on STORM data
