# Supplementary material for: Application of convolutional neural networks towards nuclei segmentation in localization-based super-resolution fluorescence microscopy images
Source: BMC Bioinformatics. 2021 Jun 15;22:325. doi: 10.1186/s12859-021-04245-x (PMC8204587; doi:10.1186/s12859-021-04245-x)
Supplement: Supplementary file 2 — Additional file 2: Figure S2. Training steps per epoch and RPN versus test accuracy. (A) Training steps per epoch versus test accuracy for Mask R-CNN and StarDist trained on the STORM images of human colon tissue dataset. Results for the cell line datasets proceeded similarly, only at higher F1-Scores. After early fluctuation, the accuracy continued to grow with steps for Mask R-CNN up to 500 steps. StarDist test accuracy, however, slowly rose to a peak at 300 steps before falling off. ANCIS did not provide a step-per-epoch variable, but provided separate training programs for its region proposal network (RPN) and segmentation algorithm. RPN epochs versus test accuracy plot (B) for ANCIS rose quickly between 0 and 50 epochs, then more slowly until reaching a peak at 600 epochs. [file 12859_2021_4245_MOESM2_ESM.pptx]

## Slide 1
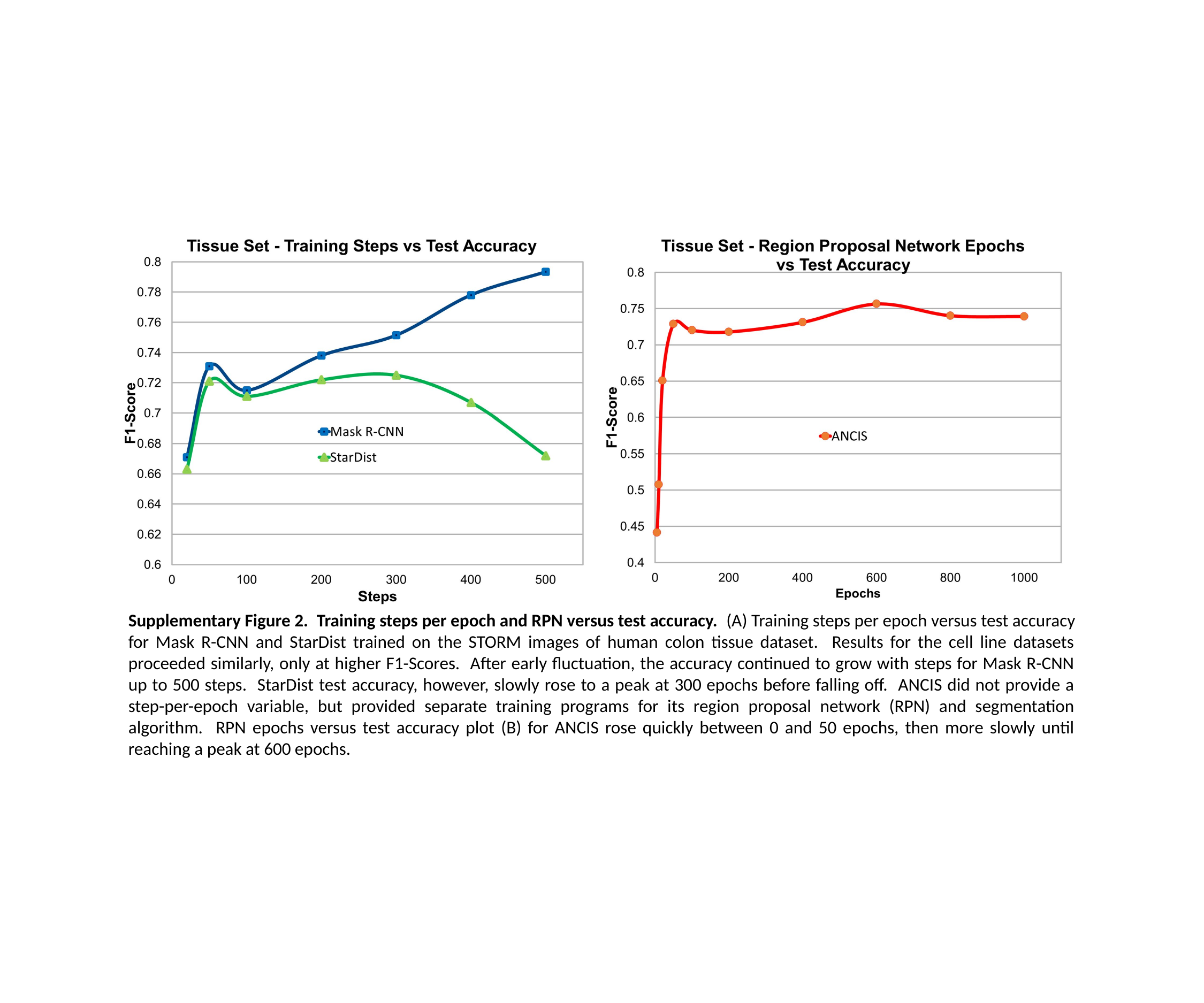

Supplementary Figure 2. Training steps per epoch and RPN versus test accuracy. (A) Training steps per epoch versus test accuracy for Mask R-CNN and StarDist trained on the STORM images of human colon tissue dataset. Results for the cell line datasets proceeded similarly, only at higher F1-Scores. After early fluctuation, the accuracy continued to grow with steps for Mask R-CNN up to 500 steps. StarDist test accuracy, however, slowly rose to a peak at 300 epochs before falling off. ANCIS did not provide a step-per-epoch variable, but provided separate training programs for its region proposal network (RPN) and segmentation algorithm. RPN epochs versus test accuracy plot (B) for ANCIS rose quickly between 0 and 50 epochs, then more slowly until reaching a peak at 600 epochs.
