# Supplementary material for: Application of convolutional neural networks towards nuclei segmentation in localization-based super-resolution fluorescence microscopy images
Source: BMC Bioinformatics. 2021 Jun 15;22:325. doi: 10.1186/s12859-021-04245-x (PMC8204587; doi:10.1186/s12859-021-04245-x)
Supplement: Supplementary file 3 — Additional file 3: Figure S3. Test accuracy versus number of images in the training set. Training set size had a distinct effect on test accuracy when conducting training for each of the three networks on the colon tissue dataset (A) and the cell line A dataset (discrete nuclear texture) (B). All networks demonstrate a general improvement in accuracy with number of images, with exceptions. In the tissue set, the Mask R-CNN test accuracy fluctuated, although the largest training set still provided the best accuracy. When training the cell line dataset, it was StarDist that fluctuated, while Mask R-CNN performed best when trained on 60 images rather than the maximum number. [file 12859_2021_4245_MOESM3_ESM.pptx]

## Slide 1
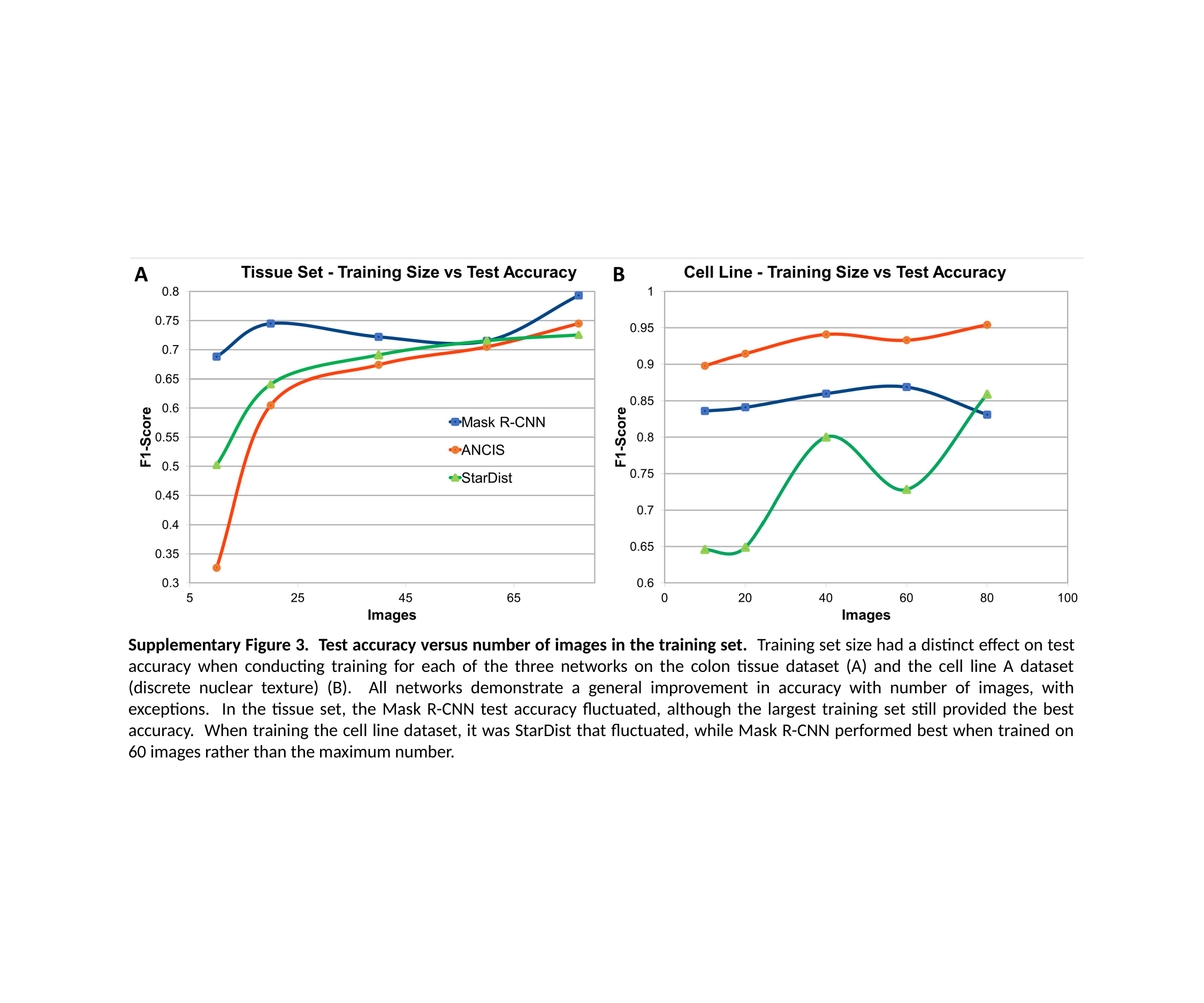

Supplementary Figure 3. Test accuracy versus number of images in the training set. Training set size had a distinct effect on test accuracy when conducting training for each of the three networks on the colon tissue dataset (A) and the cell line A dataset (discrete nuclear texture) (B). All networks demonstrate a general improvement in accuracy with number of images, with exceptions. In the tissue set, the Mask R-CNN test accuracy fluctuated, although the largest training set still provided the best accuracy. When training the cell line dataset, it was StarDist that fluctuated, while Mask R-CNN performed best when trained on 60 images rather than the maximum number.
