# Supplementary material for: Application of convolutional neural networks towards nuclei segmentation in localization-based super-resolution fluorescence microscopy images
Source: BMC Bioinformatics. 2021 Jun 15;22:325. doi: 10.1186/s12859-021-04245-x (PMC8204587; doi:10.1186/s12859-021-04245-x)
Supplement: Supplementary file 4 — Additional file 4: Figure S4. Example Mask R-CNN segmented STORM images of colon tissue at normal and various pathological states. States include (A) normal healthy tissue, (B) a low-grade dysplasia, (C) a high-grade dysplasia, and (D) an invasive adenocarcinoma. Results for the normal state were the most accurate, followed by low-grade, high-grade and invasive cancer. This demonstrates a decrease in test accuracy with decreasing nuclear cohesion. Segmentation was conducted using Mask R-CNN trained on the STORM colon tissue dataset. [file 12859_2021_4245_MOESM4_ESM.pptx]

## Slide 1
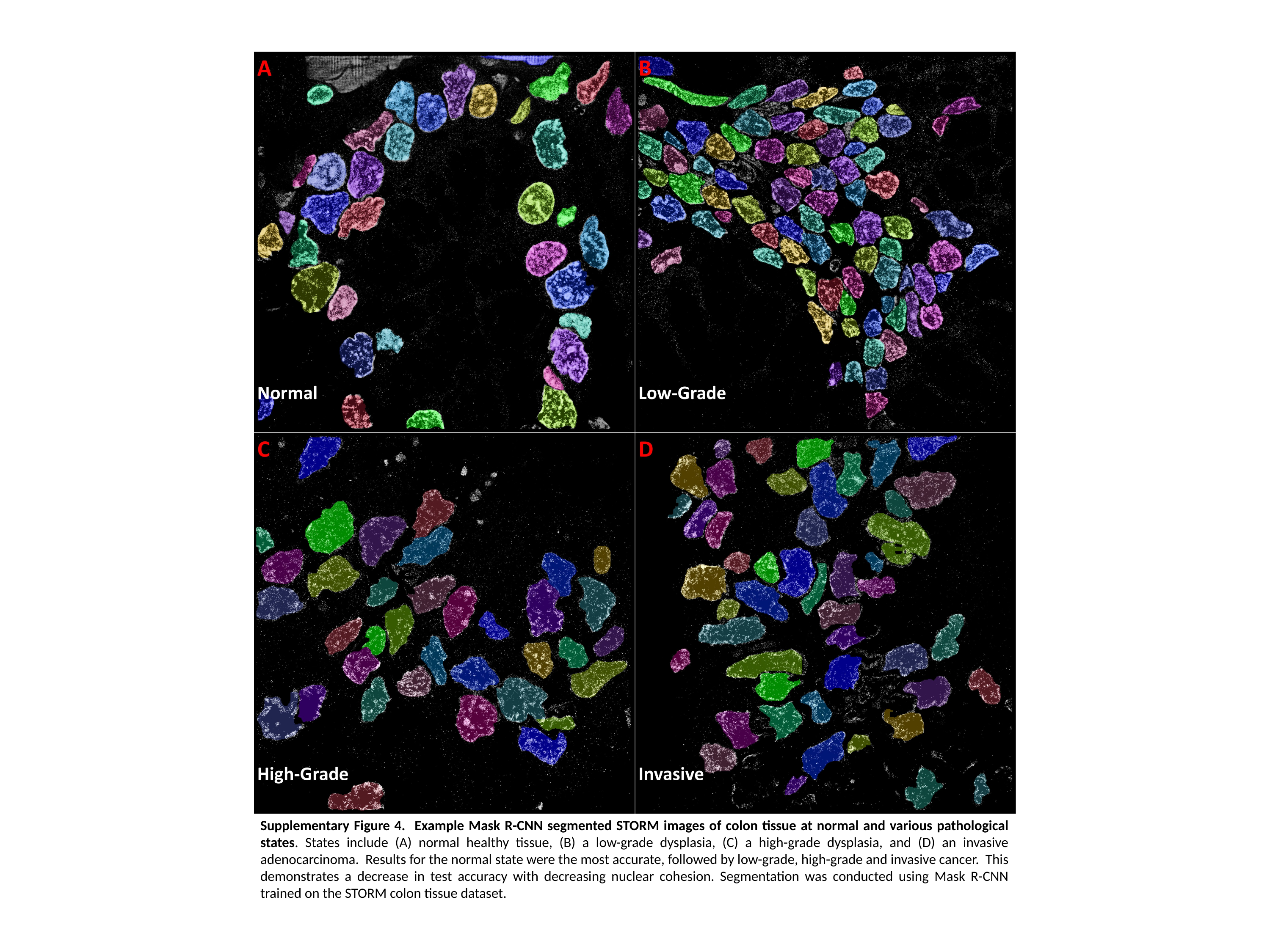

Supplementary Figure 4. Example Mask R-CNN segmented STORM images of colon tissue at normal and various pathological states. States include (A) normal healthy tissue, (B) a low-grade dysplasia, (C) a high-grade dysplasia, and (D) an invasive adenocarcinoma. Results for the normal state were the most accurate, followed by low-grade, high-grade and invasive cancer. This demonstrates a decrease in test accuracy with decreasing nuclear cohesion. Segmentation was conducted using Mask R-CNN trained on the STORM colon tissue dataset.
