# Supplementary material for: Application of convolutional neural networks towards nuclei segmentation in localization-based super-resolution fluorescence microscopy images
Source: BMC Bioinformatics. 2021 Jun 15;22:325. doi: 10.1186/s12859-021-04245-x (PMC8204587; doi:10.1186/s12859-021-04245-x)
Supplement: Supplementary file 5 — Additional file 5: Figure S5. Nuclei segmentation on STORM images from prostate tissue dataset. Segmentation of prostate tissue images using models which were pre-trained on the STORM colon tissue dataset. The original STORM image of prostate tissue (A) was segmented using (B) Mask R-CNN, (C) ANCIS and (D) StarDist networks. All three networks had multiple false negatives, however border placement appeared fairly accurate on the true positives. Mask R-CNN performed the best on this tissue dataset. [file 12859_2021_4245_MOESM5_ESM.pptx]

## Slide 1
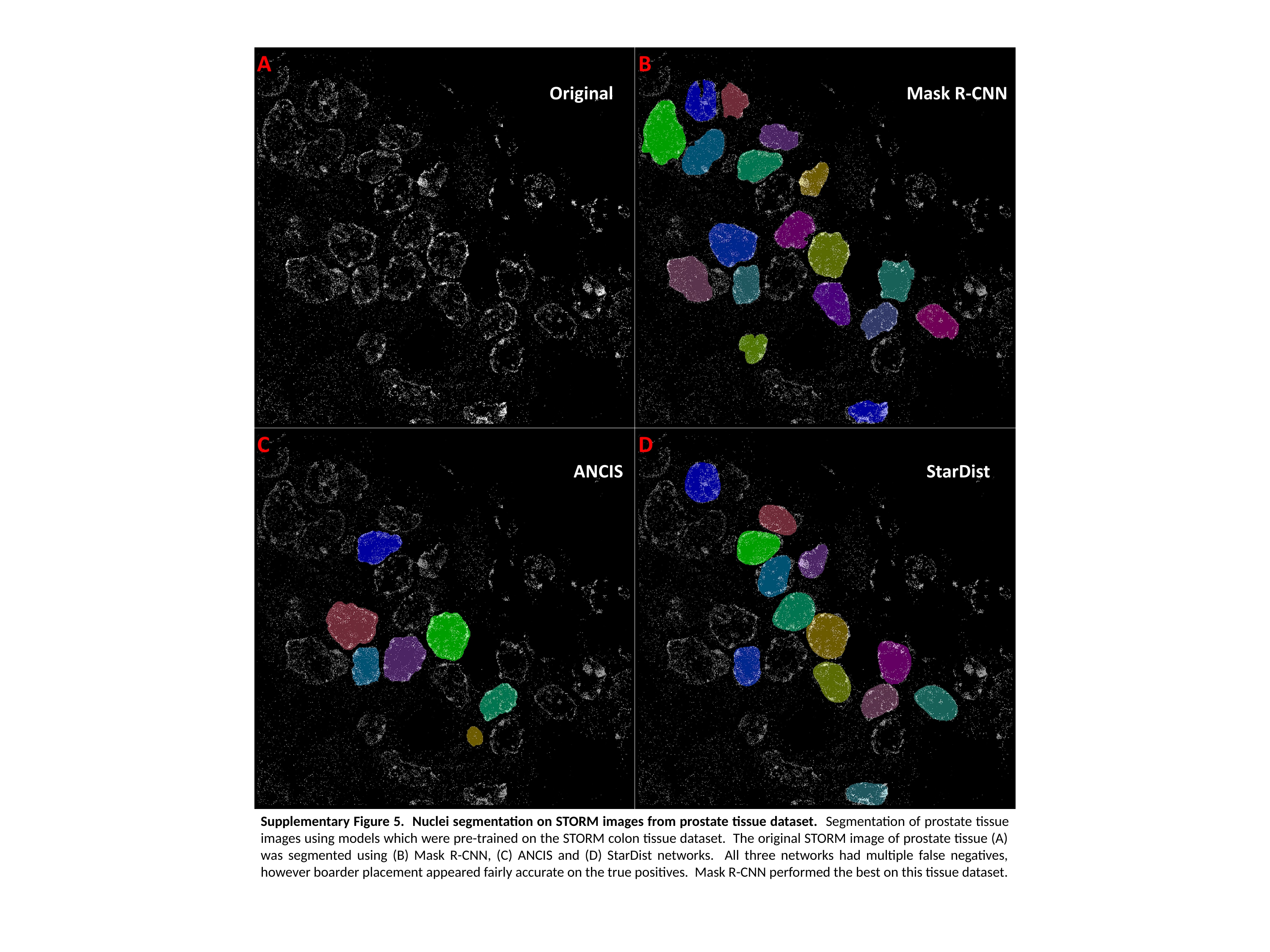

Supplementary Figure 5. Nuclei segmentation on STORM images from prostate tissue dataset. Segmentation of prostate tissue images using models which were pre-trained on the STORM colon tissue dataset. The original STORM image of prostate tissue (A) was segmented using (B) Mask R-CNN, (C) ANCIS and (D) StarDist networks. All three networks had multiple false negatives, however boarder placement appeared fairly accurate on the true positives. Mask R-CNN performed the best on this tissue dataset.
