# Supplementary material for: Application of convolutional neural networks towards nuclei segmentation in localization-based super-resolution fluorescence microscopy images
Source: BMC Bioinformatics. 2021 Jun 15;22:325. doi: 10.1186/s12859-021-04245-x (PMC8204587; doi:10.1186/s12859-021-04245-x)
Supplement: Supplementary file 6 — Additional file 6: Figure S6. Improved nuclei segmentation of StarDist method when trained on a mixed dataset. Representative nuclei segmentation of the StarDist algorithm on our cell line test images when training was conducted on our cell line A dataset (A), versus when trained on a combined dataset (B) (dataset included Kaggle, STORM colon tissue, and STORM cell line images). Similarly, we trained StarDist on our colon tissue dataset and applied the model to our colon tissue test set (C), and then applied the StarDist trained on the combined dataset to the same test set (D). Training on the combined dataset clearly improved instance border accuracy, particularly for the cell line images (B), and reduced false negatives in the tissue images (D). ANCIS demonstrated a similar improvement when trained on the combined dataset and tested on the tissue images. Mask R-CNN alone did not improve when trained on the combined dataset. [file 12859_2021_4245_MOESM6_ESM.pptx]

## Slide 1
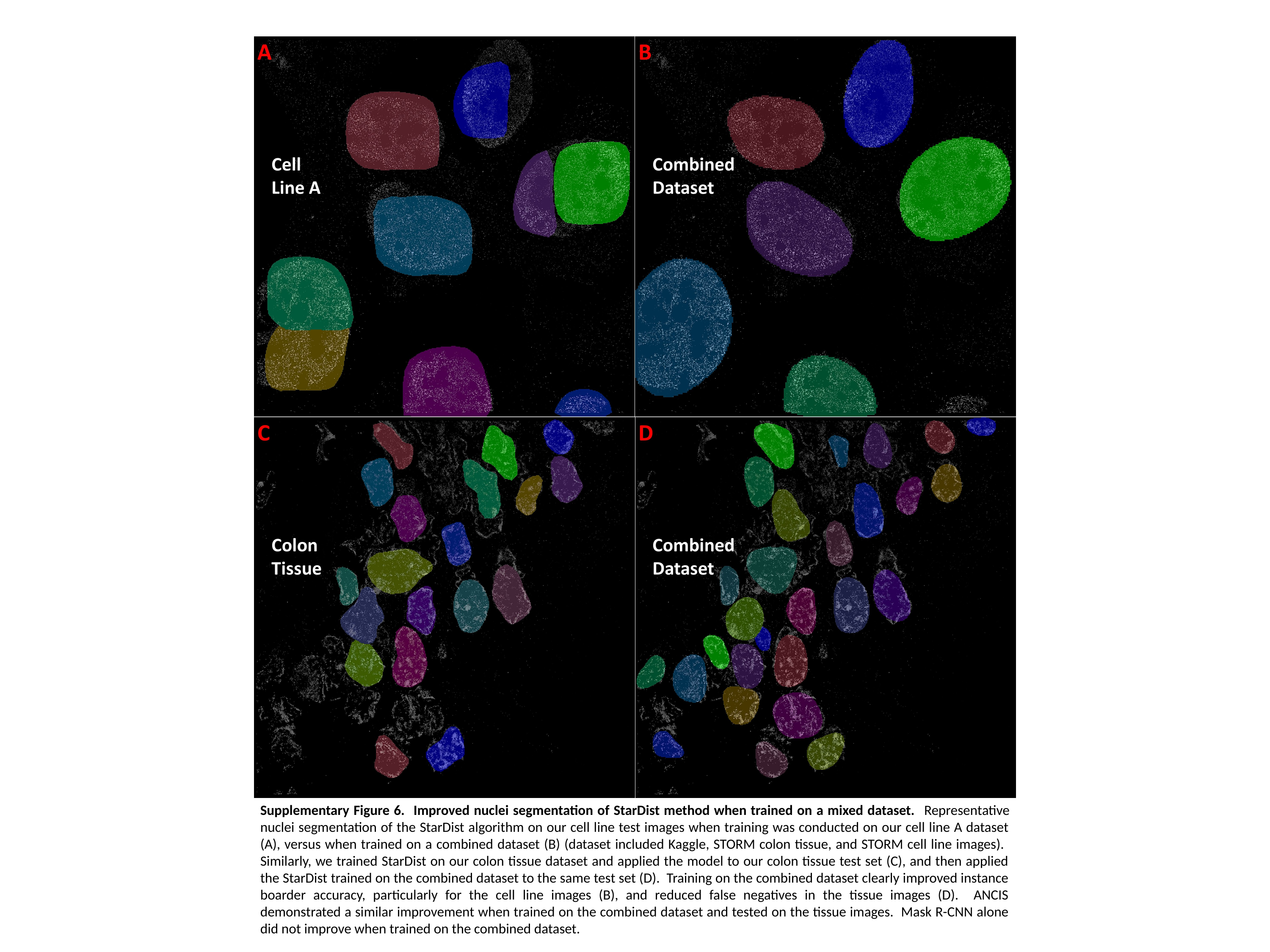

Supplementary Figure 6. Improved nuclei segmentation of StarDist method when trained on a mixed dataset. Representative nuclei segmentation of the StarDist algorithm on our cell line test images when training was conducted on our cell line A dataset (A), versus when trained on a combined dataset (B) (dataset included Kaggle, STORM colon tissue, and STORM cell line images). Similarly, we trained StarDist on our colon tissue dataset and applied the model to our colon tissue test set (C), and then applied the StarDist trained on the combined dataset to the same test set (D). Training on the combined dataset clearly improved instance boarder accuracy, particularly for the cell line images (B), and reduced false negatives in the tissue images (D). ANCIS demonstrated a similar improvement when trained on the combined dataset and tested on the tissue images. Mask R-CNN alone did not improve when trained on the combined dataset.
