# Supplementary material for: Application of convolutional neural networks towards nuclei segmentation in localization-based super-resolution fluorescence microscopy images
Source: BMC Bioinformatics. 2021 Jun 15;22:325. doi: 10.1186/s12859-021-04245-x (PMC8204587; doi:10.1186/s12859-021-04245-x)
Supplement: Supplementary file 7 — Additional file 7: Figure S7. Improper noise model can result in nuclei deletion when denoising. When conducting denoising using an improperly trained UNet noise model (trained only on dense noise), less noisy super-resolution images from our colon tissue dataset (A), which also contain less densely labeled nuclei, sometimes suffered from nuclei deletion (C) due to the networks inability to accurately distinguish noise from cell data (B). The noise probability map (B) from the UNet model shows a high probability of suspected noise (white regions) over several nuclei sites. The resulting denoised image, when this model is applied to the original image (A), shows the deletion of real nuclei (C). [file 12859_2021_4245_MOESM7_ESM.pptx]

## Slide 1
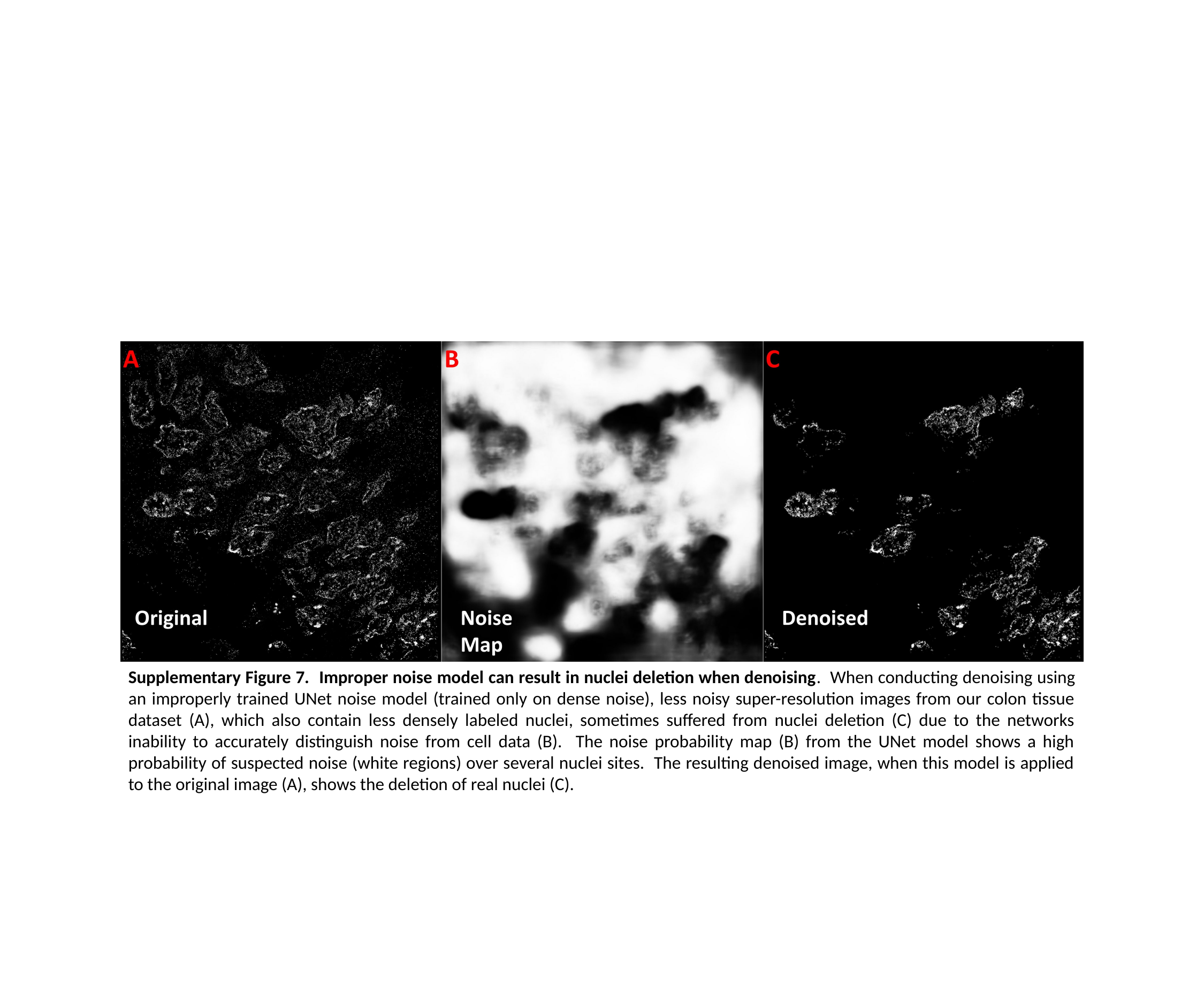

Supplementary Figure 7. Improper noise model can result in nuclei deletion when denoising. When conducting denoising using an improperly trained UNet noise model (trained only on dense noise), less noisy super-resolution images from our colon tissue dataset (A), which also contain less densely labeled nuclei, sometimes suffered from nuclei deletion (C) due to the networks inability to accurately distinguish noise from cell data (B). The noise probability map (B) from the UNet model shows a high probability of suspected noise (white regions) over several nuclei sites. The resulting denoised image, when this model is applied to the original image (A), shows the deletion of real nuclei (C).
