# Supplementary material for: Application of convolutional neural networks towards nuclei segmentation in localization-based super-resolution fluorescence microscopy images
Source: BMC Bioinformatics. 2021 Jun 15;22:325. doi: 10.1186/s12859-021-04245-x (PMC8204587; doi:10.1186/s12859-021-04245-x)
Supplement: Supplementary file 8 — Additional file 8: Table S1. Cell types included in our STORM datasets, along with their respective labeled targets, biological states, fluorophores and datasets. [file 12859_2021_4245_MOESM8_ESM.pptx]

## Slide 1
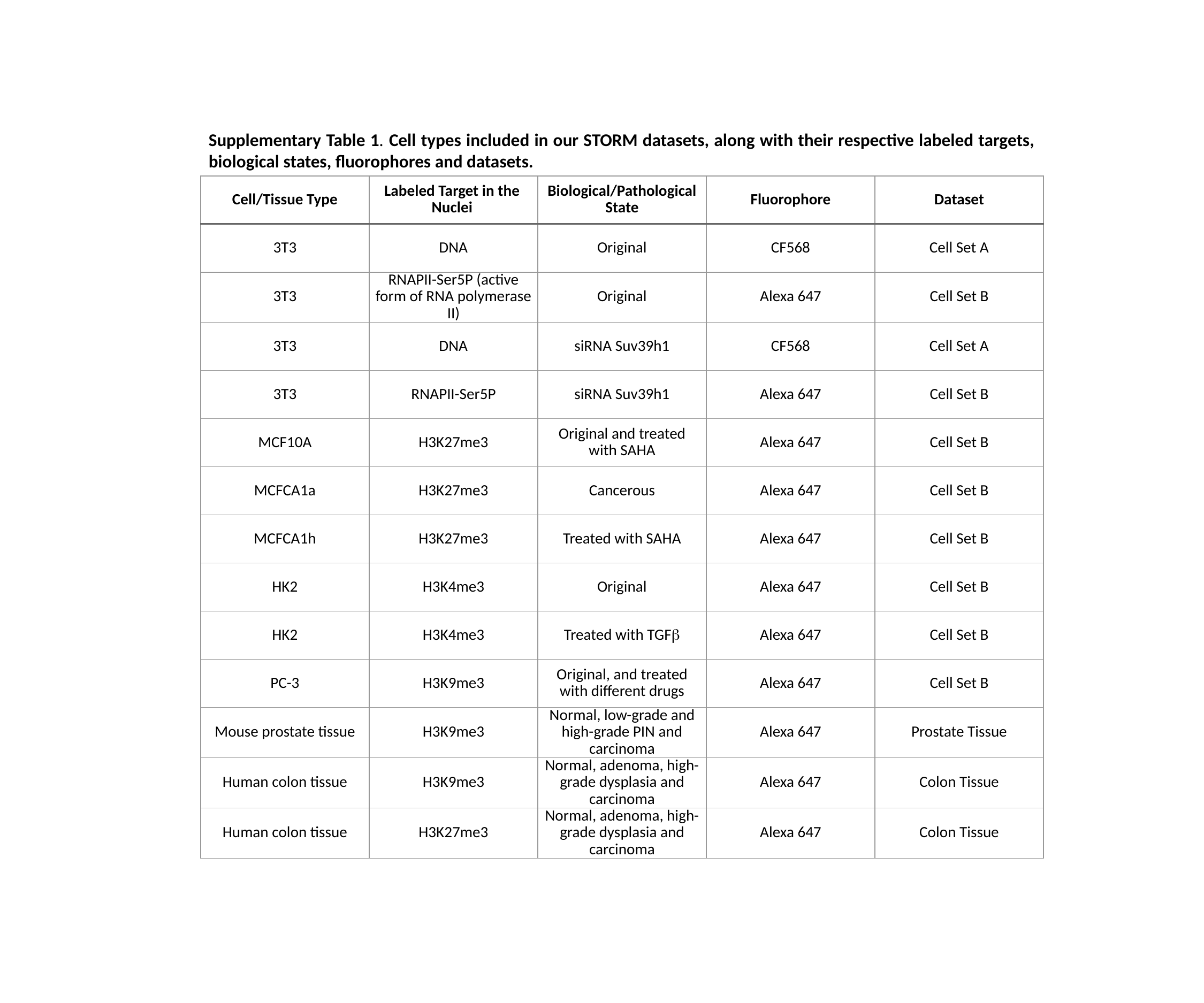

Supplementary Table 1. Cell types included in our STORM datasets, along with their respective labeled targets, biological states, fluorophores and datasets.
| Cell/Tissue Type | Labeled Target in the Nuclei | Biological/Pathological State | Fluorophore | Dataset |
| --- | --- | --- | --- | --- |
| 3T3 | DNA | Original | CF568 | Cell Set A |
| 3T3 | RNAPII-Ser5P (active form of RNA polymerase II) | Original | Alexa 647 | Cell Set B |
| 3T3 | DNA | siRNA Suv39h1 | CF568 | Cell Set A |
| 3T3 | RNAPII-Ser5P | siRNA Suv39h1 | Alexa 647 | Cell Set B |
| MCF10A | H3K27me3 | Original and treated with SAHA | Alexa 647 | Cell Set B |
| MCFCA1a | H3K27me3 | Cancerous | Alexa 647 | Cell Set B |
| MCFCA1h | H3K27me3 | Treated with SAHA | Alexa 647 | Cell Set B |
| HK2 | H3K4me3 | Original | Alexa 647 | Cell Set B |
| HK2 | H3K4me3 | Treated with TGFβ | Alexa 647 | Cell Set B |
| PC-3 | H3K9me3 | Original, and treated with different drugs | Alexa 647 | Cell Set B |
| Mouse prostate tissue | H3K9me3 | Normal, low-grade and high-grade PIN and carcinoma | Alexa 647 | Prostate Tissue |
| Human colon tissue | H3K9me3 | Normal, adenoma, high-grade dysplasia and carcinoma | Alexa 647 | Colon Tissue |
| Human colon tissue | H3K27me3 | Normal, adenoma, high-grade dysplasia and carcinoma | Alexa 647 | Colon Tissue |
