# Supplementary material for: Application of convolutional neural networks towards nuclei segmentation in localization-based super-resolution fluorescence microscopy images
Source: BMC Bioinformatics. 2021 Jun 15;22:325. doi: 10.1186/s12859-021-04245-x (PMC8204587; doi:10.1186/s12859-021-04245-x)
Supplement: Supplementary file 9 — Additional file 9: Figure S8. Example cell line images, each expressing different textures. Textures include a dim, diffuse pattern (active form of RNA polymerase II in 3T3 cells) (A), more densely placed labels (DNA in 3T3 cells) (B) and (H3K4me3 in HK2 cells) (C), and discrete labels (H3K27me3 in CA1h cells) (D). [file 12859_2021_4245_MOESM9_ESM.pptx]

## Slide 1
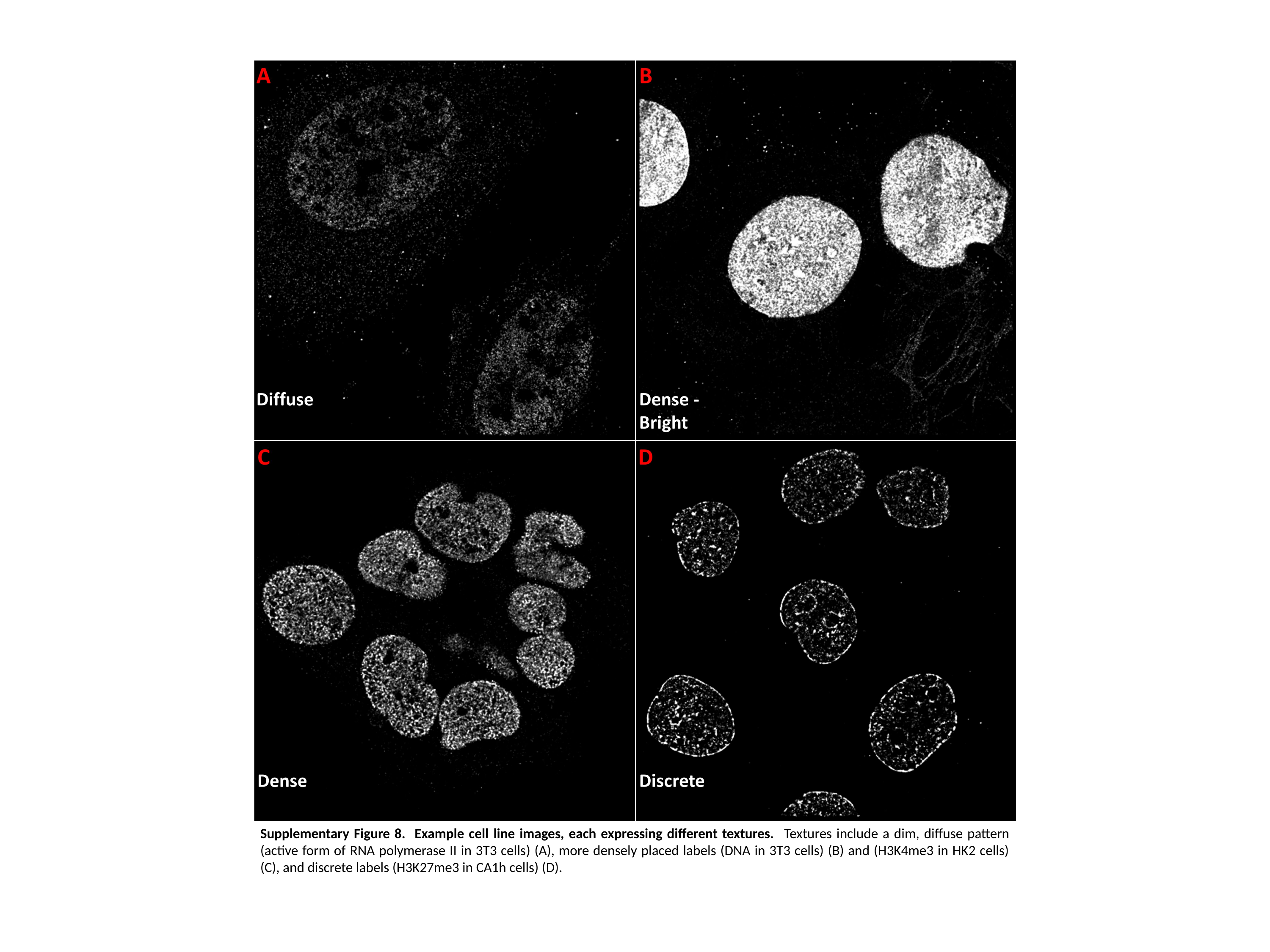

Supplementary Figure 8. Example cell line images, each expressing different textures. Textures include a dim, diffuse pattern (active form of RNA polymerase II in 3T3 cells) (A), more densely placed labels (DNA in 3T3 cells) (B) and (H3K4me3 in HK2 cells) (C), and discrete labels (H3K27me3 in CA1h cells) (D).
