# Supplementary material for: Application of convolutional neural networks towards nuclei segmentation in localization-based super-resolution fluorescence microscopy images
Source: BMC Bioinformatics. 2021 Jun 15;22:325. doi: 10.1186/s12859-021-04245-x (PMC8204587; doi:10.1186/s12859-021-04245-x)
Supplement: Supplementary file 10 — Additional file 10: Figure S9. Tiling method example with and without overlap. When the test image is divided into non-overlapping squares, or tiles, for segmentation, the borders between tiles will act as artificial borders between labeled detected instances upon image reconstruction, creating divided predictions (upper diagram). Overlapping allows for a post-segmentation program to sample the overlapping region in adjacent tiles and merge overlapping instances during reconstruction (lower diagram). [file 12859_2021_4245_MOESM10_ESM.pptx]

## Slide 1
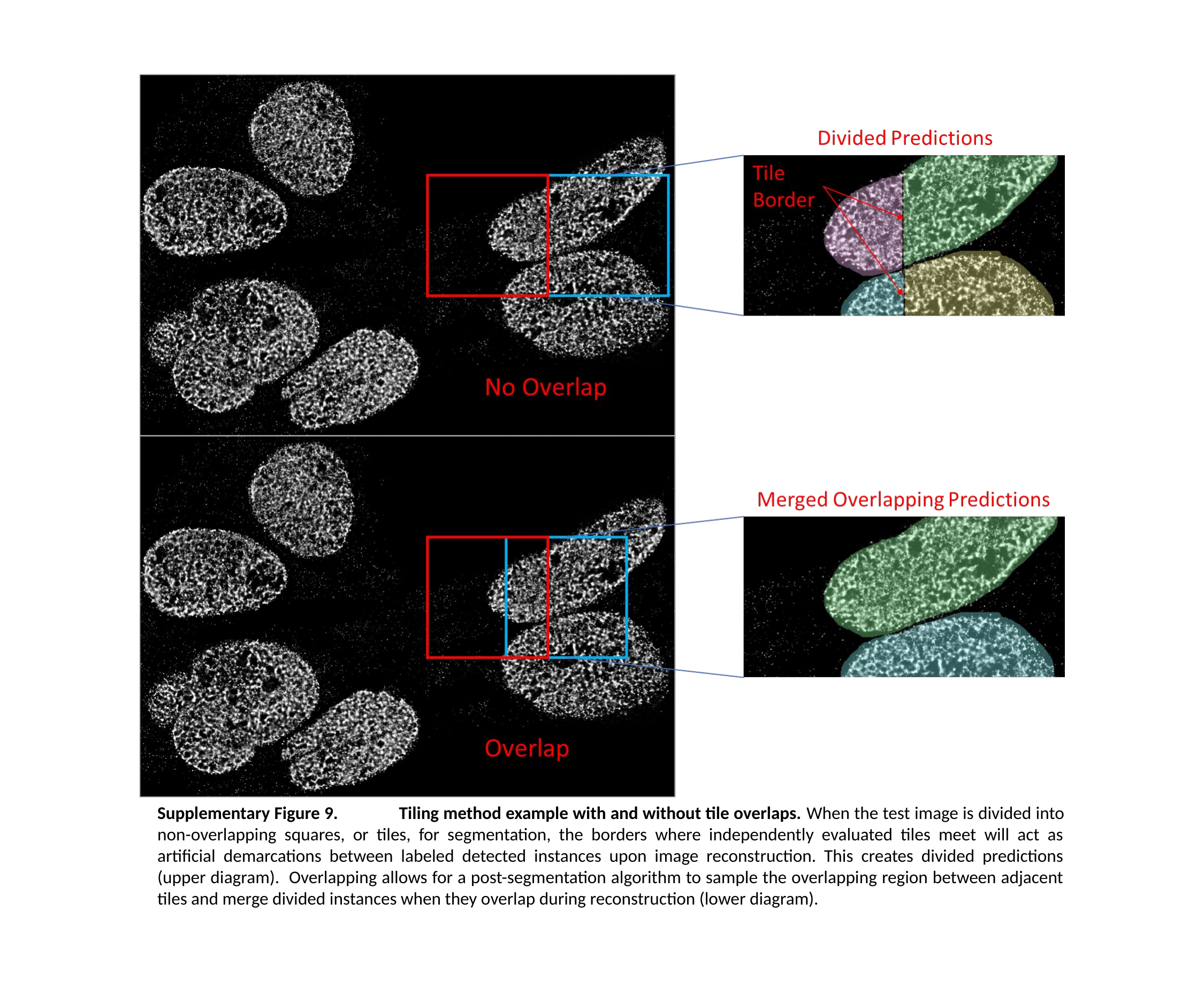

Supplementary Figure 9.	Tiling method example with and without tile overlaps. When the test image is divided into non-overlapping squares, or tiles, for segmentation, the borders where independently evaluated tiles meet will act as artificial demarcations between labeled detected instances upon image reconstruction. This creates divided predictions (upper diagram). Overlapping allows for a post-segmentation algorithm to sample the overlapping region between adjacent tiles and merge divided instances when they overlap during reconstruction (lower diagram).
